# Perception of phage therapy and research across selected professional and social groups in Poland

**DOI:** 10.3389/fpubh.2025.1490737

**Published:** 2025-02-11

**Authors:** Maciej Żaczek, Marcin W. Zieliński, Andrzej Górski, Beata Weber-Dąbrowska, Ryszard Międzybrodzki

**Affiliations:** ^1^Bacteriophage Laboratory, Department of Phage Therapy, Hirszfeld Institute of Immunology and Experimental Therapy, Polish Academy of Sciences, Wrocław, Poland; ^2^The Centre of Sociological Research, Institute of Philosophy and Sociology, Polish Academy of Sciences, Warsaw, Poland; ^3^Phage Therapy Unit, Hirszfeld Institute of Immunology and Experimental Therapy, Polish Academy of Sciences, Wrocław, Poland; ^4^Department of Clinical Immunology, Medical University of Warsaw, Warsaw, Poland

**Keywords:** bacteriophages, phage therapy, public perception, social groups, experimental treatment, antibiotic resistance, survey, health care

## Abstract

There is no doubt that in the last 15 years phage therapy has re-emerged from the shadow of antibiotics, from the perspective of both scientists and various patient advocacy groups. Despite some important progress, there is little to no data on phage therapy perceptions in key groups, i.e., patients and their relatives, physicians and anyone who could potentially become infected with antibiotic-resistant bacteria. To the best of our knowledge, with 1,098 collected questionnaires, this article constitutes the first large-scale analysis on phage therapy perceptions, interest and knowledge among ordinary people in a country with a long, internationally recognized, tradition of treating patients with phages. In addition to 36 general questions addressed to everyone (including lay people), representatives of the health care sector and science and research sector received individually selected questions. Further, each participant had a chance to take part in a short quiz (consisting of 12 questions) verifying their basic knowledge about bacteriophages, their history, biology and therapeutic connotations. Awareness of antibiotic resistance was very high (above 90%) but contradicted the low level of knowledge about associated risks (12%). Consciousness of phage therapy varied between 8.9% (people taking care of household chores as their primary activity) to 37.7% (people with higher education) and 39.7% (inhabitants of large cities) while the readiness to use such treatments was very high (84.4%) despite the need to pay for it. The level of awareness of bacteriophages and phage therapy was clearly correlated with the acceptance of this type of treatment and the social acceptance to allocate further funds for the development of phage research. Interestingly, physicians were quite reluctant to deepen their knowledge in the field of phage therapy with just over one third (37.5%) ready to do so. With the COVID-19 pandemic in the background, we also explored how the pandemic influenced the interest in experimental therapies in general, which makes this article a potential universal compendium on perceptions of experimental therapies in the coming years.

## 1 Introduction

According to Earl Babbie, an American sociologist and author of many books in the field of social research, some paradigms appear obvious to the public and almost no one tries to discuss the roots of such perception (1). People tend to take things for granted but most of what we know is just an assumption or the result of faith. By analogy, the positive climate around phage treatment and its rapid global development has provoked a favorable perception of such a form of treatment among those who have or might have contact with it, including lay people. The potential of such an encouraging campaign for phage therapy was discussed by Hausler in a suggestively entitled article *Phages Make for Jolly Good Stories* (2). The number of appealing statements about phage therapy coming from the professionals, who tend to present it mainly in a positive light makes us think that people's perception of such novel therapy should be the least of our concern (3–8). However, has anyone ever tried to investigate this at a deep level? Recent events related to the COVID-19 pandemic have shown a quite surprising picture, in which even life-threating situations have not prevented the anti-vaccine movement from entering the mainstream. The problem has reached such proportions that even the World Health Organization labeled vaccine hesitancy as a major public health threat (9). By analyzing the history of the anti-vaccine movement, one might draw the conclusion that the current situation is the exact opposite of what most of us thought: the greater the incentives for vaccination from health care authorities, the greater the resistance from the public (10, 11). The complexity of social behavior in terms of human health was highlighted in 2020 by the U.S. Department of Health and Human Services by admitting that health and health-related behaviors are determined by influences at multiple levels, including personal (biological, psychological), organizational/institutional, environmental (both social and physical) and political levels (12). Similar conclusions were drawn by Charani et al. who saw major challenges in building health awareness in sociocultural disparities such as, among others, race, ethnicity, gender identity and cultural as well as religious practices or even migration status and geographical influence on health (13). For instance, it has been observed that smoking among adolescents is inevitably associated with similar habits in peers from the same environment, but this does not necessarily extend to quitting smoking (12). Such dependence could be a possible explanation for the above-mentioned vaccine hesitancy in particular social groups, for instance, ethnic minority groups (14).

Regardless of the above, phage therapy presents a rather unusual case in the context of public health. First, its public perception is still relatively unknown. Although phage therapy is becoming more and more popular, one cannot say that patients are persuaded to use this form of treatment as was the case on a global scale for vaccinations during the COVID-19 pandemic. Mostly, patients with infections caused by antibiotic-resistant (ABR) bacteria seek help on their own. Such an approach, where patients seek phage treatment by themselves, was recently emphasized by other authors (15). This is certainly a common situation in the Phage Therapy Unit (PTU) established at the Hirszfeld Institute of Immunology and Experimental Therapy (HIIET) in Wrocław, Poland (16). Moreover, following screening procedures, patients often do not qualify for such treatment. Over the 18 years of operation of PTU, less than half of the patients (nearly 823) qualified for phage treatment out of 2,286 patients registered at the PTU (as of December 2023) (16). In Belgium, between 1 January 2008 and 30 April 2022, 1,066 phage therapy requests were submitted to the Queen Astrid Military Hospital resulting in 100 patients treated with phages (17). Another determinant that makes the phage therapy landscape different from the COVID-19 pandemic, particularly in Poland, is the lack of successful clinical trials (due to pharma industry's hesitancy) and the lack of non-commercial clinical trials. Putting together the first non-commercial clinical trial in Poland was commenced at PTU in 2022 thanks to the funding from the Polish Medical Research Agency. It is worth mentioning that until a few years ago non-commercial clinical trials were almost non-existent in Poland (18). The main difference that could possibly influence the public's perception of phage treatment, contrary to the COVID-19 example, is the authorization process used for vaccines against SARS-CoV-2. Such a process is required by health authorities before any new drug is released to the market. We have all witnessed a historically rapid approval process for emergency use on an unprecedented scale, especially in the case of two mRNA-based COVID-19 vaccines (19). Such haste aroused distrust from a large part of the society. The following occurrence of adverse events, so-called *adverse event following immunization* (AEFI), which led to the suspension of certain vaccines in some countries, unequivocally increased public concern and perception of such treatment to an even greater extent (20, 21). Contrary to the rapid approval of new COVID-19 vaccines, phage therapy until recently encountered difficulties at a basic level (for instance, how to classify the natural bacteriophages in terms of medicinal products) even though antibiotic resistance is a major twenty-first century health challenge (22–26). In addition, adverse events following the administration of phage preparations are rare, mostly mild and, as mentioned above, phage therapy has never been the subject of acute safety concerns, including those discussed in the mainstream (16, 22, 27).

From the above description, a picture emerges suggesting that extensively focusing on phage therapy outcomes and legislative obstacles almost completely minimized the need for social research conducted in groups of people differing from each other, among others, by gender, age, education, employment and material status or experience with health care they received. There are only a few articles that have investigated people's opinions on phage therapy with a relatively limited sample size (28, 29). The need to investigate public understanding of phage therapy and research was recently emphasized by Hitchcock et al. (30). The authors believe that successful commencement and development of phage treatment in a clinical setting must be combined with listening to people about their concerns, experiences and expectations. Followed by a similar belief, we investigated how this situation looks in Poland, a country that has the largest in the EU database of patients who have undergone phage therapy (over 750 cases) in the form of compassionate treatment in accordance with current ethical and legal requirements (31).

There was an expectation that phage therapy and phage research awareness remain at the low level in Poland. Such assumptions were based on our long-term observation of the number of mainstream publications dealing with phage therapy and the time devoted to this topic in the Polish media. PTU, being the only phage therapy center in Poland and one of only a few across the globe, is not able to attract such attention as major oncology or cardiology centers to which all involved parties, i.e., the pharmaceutical industry, health care institutions, regulatory agencies, media and patients devote a lot of attention. In addition, recent investigation by McCammon et al. conducted with nearly 800 respondents clearly showed that phage therapy remains poorly understood by the public in the UK (32). The aim of the article was to present the general level of knowledge and perception of phage therapy and research in Polish society. To our knowledge this paper is the first analysis to be conducted on such a large group of people (over 1,000 individuals) regarding perception and understanding of phage research and therapy in humans.

## 2 Methodology

### 2.1 Overview

Surveys in health care must adhere to the same rules as any other typical questionnaire and the authors face similar problems that may affect the results, e.g., responders may wish to manipulate the answers, questions might be too subjective or the sample size too low (33). Since we encountered all these issues while working on our survey, it was important to spread the survey over time in order to maximize the potential of obtaining the most diverse and large group of respondents who understand the questions being asked. Due to the complex structure of our questionnaire consisting of up to 70 questions (Q), including conditional ones that were available to respondents depending on their previous answers, the number of social groups addressed in it and limited financial support, we decided to exploit a web survey. Such an approach is not only cheaper and faster than standard solutions (like mail-based polls) but also their popularity has consistently grown in recent years (34). We were aware that such an approach was not perfect, however, as it required access to the Internet, which to some extent excluded the less affluent as well as the older adults and disabled groups. Moreover, a less personalized approach typical in a web-based environment carried the risk of a reduced response from potential respondents, as confirmed by other authors (35, 36). Finally, given the scientific nature of the survey, there was a risk of general misunderstanding of the topic and terminology used across questions which could lead to incorrect answers, or the questionnaire not being completed (37). Nonetheless, a web survey was chosen as a methodologically acceptable way of conducting this kind of research.

### 2.2 Survey structure

Our questionnaire was prepared in two identical versions, separately for women and men, which was caused solely by grammatical complexities in Polish. The decision on which version of the questionnaire to use was made by the respondent at the very beginning, without the possibility of changing the choice later. A short description, invitation and a “thank you” message for the willingness to participate in the survey was placed on a dedicated website using HIIET servers. The collection of questionnaires started on 13 June 2022 and lasted until 18 April 2023 (data cut-off), i.e., for 10 months and 5 days. A link to the above-mentioned website (*hirszfeld.pl/ankieta*) was the master link sent to potential participants. The link led to a survey located on the servers of the Institute of Philosophy and Sociology, Polish Academy of Sciences in Warsaw, Poland. The questionnaire was completed anonymously and voluntarily. The information gathered did not put the participants at risk in any form. Further, they were able to end the questionnaire at any time, although this resulted in the loss of previously provided answers and the need to start the process from the beginning (if such a decision was made). Of note, if the questionnaire was not fully completed, the system did not consider it valid.

An original, structured, cross-sectional, self-administered questionnaire was designed with the CAWI (Computer Assisted Web Interview) technique, i.e., a questionnaire in which the respondent is asked to complete a questionnaire in electronic form. Among 70 single or multiple-choice questions, 36 were addressed to all respondents (first module), 11 were intended for health care professionals (second module) and 10 for people working in the scientific and research sector (third module). Persons declaring work in both above-mentioned sectors had to take part in both modules. It should be added that depending on the answers given, access to some subsequent questions was restricted. The last part, optional for each survey participant, was based on completing a quiz consisting of 12 questions about phage history, biology and phages' therapeutic connotations (fourth module). Although answering questions in the quiz required a bit more knowledge than the average, we had in mind when creating these questions that the level of difficulty should be adapted to lay people who may have never heard of phage therapy. Therefore, each question in the quiz offered the option to provide the answer *I don't know* in order to avoid randomly selected answers. Our goal was to examine the real level of understanding in the subject of phage therapy. We did not want to cause respondents to feel embarrassed or ashamed due to a lack of sufficient knowledge. The possibility of choosing the answer *I don't know* was intended to legitimize the lack of any proficiency in the examined matter. In addition, there was a separate question in which we asked for consent to participate in quiz. Thus, an active consent was required. At this point it should be added that the answers from the first three modules were still valid even if a respondent chose not to take the quiz (fourth module), which was communicated to each person before making the decision. The entire questionnaire with a full list of questions and answer options is provided in Appendix 1. Because English grammar is characterized by gender-neutral second person pronouns, there was no need to provide both versions of the questionnaire (for women and men) that was used in our research.

### 2.3 Recruitment of potential respondents

Seeking potential participants was probably the most challenging task we had to deal with. In the following weeks we started creating mailing lists based on publicly available contact details asking people, mostly professionals from health care and scientific sector, to fill out questionnaires. Such a request was accompanied by a brief explanation of the purpose of the survey. Each time (except for patient advocacy groups in which case e-mails were sent directly by the authors) the official email request was signed by the HIIET's authorities. A full list of entities we have contacted across Poland is enclosed in the Appendix 2. Shortly we reached 11 patients' associations, 67 institutes of the Polish Academy of Sciences (i.e., all existing institutes in Poland), 43 health care entities as well as 62 private and public universities. In addition, we spread the message across the official website of HIIET PAS, BINWIT social media channels (Facebook, ResearchGate, Twitter), we next reached out to our relatives, friends, colleagues and fellow workers. We were fully aware that such a strategy presented a lack of appropriate representativeness of the group hence this period was considered the first part of questionnaire collection. The increase in the number of completed questionnaires was tracked at irregular weekly, bi-weekly or monthly intervals (separately for male and female version of the survey) with the first follow-up on 22 September 2022, i.e., three and a half months after the survey was launched. A summary that gives a general idea of the number of responses we received after each mail distribution is shown in Supplementary Table 1.

After reaching half of the target number of respondents (~500) and an initial analysis of the results suggesting an expected lack of sufficient diversity we initiated cooperation with an experienced Warsaw-based research agency SW Research (*swresearch.pl*) whose clients include the world's largest brands. SW Research has completed over 3,600 research projects and is a signatory to the Interviewers' Work Quality Control Program as well as a member of the European Society for Opinion and Marketing Research (ESOMAR). The company's task was to provide access to its online panel of respondents (about 400 people, half women and men), which was assumed to be a group less associated with professionals and more representative of Polish society than the panel of people (more or less familiarized with the problem of antibiotic resistance and phage therapy) we reached on our own, hereinafter called Arm A. This group of interviewees received a form of compensation for completing the questionnaire directly from and on the terms of SW Research. Respondents from the SW Research database (hereinafter called Arm B) completed our survey between 10–14 March and 7–11 April 2023. In order to eliminate unreliable respondents from the analysis, questionnaires completed by respondents within the time frame belonging to the first decile were excluded. It ranged from 1 min 11 s to 5 min and 19 s. In the authors' opinion, this time was insufficient to provide thoughtful answers to all questions, even in the shortest version of the questionnaire.

### 2.4 Analysis design

The categorical variables were reported as frequencies and/or percentages and compared by sex, age, place of residence, level of education, employment status, experience with health care, profession and financial status. Both aforementioned datasets, Arm A and the Arm B, were analyzed jointly except for the key questions regarding knowledge and perception of phage therapy and research (due to expected significant differences among these two groups). The core design of analysis is presented in Supplementary Table 2.

### 2.5 Statistics

The data collected from both questionnaires (female and male versions) were merged into one file and analyzed using STATISTICA v. 13.3 (TIBCO Sotfware Inc., Palo Alto, CA, USA). Some calculations were made using Excel (Microsoft), version 16.72 for MacOS as weel as GNU PSPP Statistical Analysis Software (version 1.6.2 for MacOs; available at https://www.gnu.org/software/pspp/). GNU PSPP is an open-source software for analysis, intended as a free alternative for IBM SPSS Statistics while both are widely used in survey research (12, 37–40). Numbers and percentages were calculated for nominal and ordinal variables and included in contingency tables. It must be emphasized that despite our efforts the sample tested was neither random nor representative, hence all parametric tests based on the randomness of the sample must have been excluded from further analysis. Pearson's chi-square test was used to assess the homogeneity of distributions. Medians and quartile ranges were calculated for the ranked variables, and the significance of differences in the two groups was verified using the Mann-Whitney U test. In the case of three or more groups, the Kruskal-Wallis test was used. *Post-hoc* analysis was done using the Dunn's test. Spearman's rank correlation coefficients (*r*_*S*_) were calculated to assess the strength and direction of correlation between two ordinal variables. To determine whether two proportions are different from each other a two-proportion Z-test was used. In all statistical tests results were considered significant if *p* < 0.05.

To assess the level of knowledge on bacteriophages and phage therapy, the author's General Knowledge about Bacteriophages scale (GKB-12) was built based on the responses to Q59–70 and used after its validation. The value of the Cronbach's α reliability index was estimated.

## 3 Results

### 3.1 Characteristics of the sample size used in the study

The overall sample size for this study was 1,098 evaluable questionnaires after removing 121 cases from 1,219 collected questionnaires due to too short response time as described above. 681 questionnaires (62%) were collected as part of our efforts through mailing lists, social media, verbal requests (Arm A) while 417 questionnaires (38%) were collected from SW Research respondents (Arm B). As for the quiz, which was optional for everyone who completed the questionnaire, 1,020 individuals (92.9%) agreed to participate (614 from Arm A and 406 from Arm B).

From the beginning of the collection of the questionnaires, women filled out them over twice as often as men, regardless of the professional groups to which we distributed our requests which is a typical occurrence observed by other authors (41, 42). However, it must be emphasized that the difference between the number of women and men was even greater in Arm A (485 women vs. 196 men) while the Arm B was initially designed to collect the same number of both genders (ultimately there were 209 women and 208 men). The majority of participants lived in Poland which should not be surprising considering the language used in the survey. However, due to a significant number of Poles living and working abroad we decided to provide an additional answer option (i.e., living outside of Poland). Nearly half of the tested population (49.1%) lived in cities above 250,000 inhabitants, 65.4% whom had completed a higher education (from a bachelor's or engineer degree onwards) and the majority (66.5%) worked as full-time employees. Over three-quarters of the recorded answers reported a *rather satisfactory* or *definitely satisfactory* financial status and almost half of the participants was satisfied with the health care they received. Clearly, the surveyed population does not reflect the Polish society, and we suspect that it does not reflect the society of any country in the European Union or a country with a developed economy. For instance, according to Statistics Poland, Poland's principal government institution responsible for statistics and census data, the real number of Poles with completed higher education is twice as small (23.1% in 2021) (43). Further, it is easy to calculate that 11 Polish cities with more than 250,000 inhabitants account for <20% of the population (compared to 49.1% in our study). The main characteristics of the examined population is shown in Table 1.

**Table 1:** The main characteristics of the examined population (1,098 individuals).

**Characteristics**	**Categories**	**Number**	**Percentage of total^a^**
Sex	Female	694	63.2%
	Male	404	36.8%
Age	≤18 y/o	56	5.1%
	19–30 y/o	247	22.5%
	31–40 y/o	260	23.7%
	41–50 y/o	244	22.2%
	51–60 y/o	179	16.3%
	>60 y/o	112	10.2%
Country of residence	Poland	1,078	98.2%
	outside Poland	20	1.8%
Place of residence	Village	213	19.4%
	City <50,000 inhabitants	165	15.0%
	City 50,000–250,000 inhabitants	181	16.5%
	City >250,000	539	49.1%
Level of education	Unfinished elementary school	2	0.2%
	Completed elementary school	58	5.3%
	Completed secondary school	7	0.6%
	Completed basic vocational school^a^	48	4.4%
	Completed high school without diploma^b^	32	2.9%
	Completed high school with diploma^c^	179	16.3%
	Completed post-high school education	51	4.6%
	Engineer or bachelor's degree	82	7.5%
	Master's degree	403	36.7%
	PhD degree	155	14.1%
	Habilitation^d^	55	5%
	Full professor	23	2.1%
	Other^e^	3	0.3%
Employment status^f^	Full-time employee	730	66.5%
	Part-time employee	75	6.8%
	Temporary, seasonal, commissioned work	93	8.5%
	Parental/maternity leave	12	1.1%
	Unemployed	33	3%
	Retired	101	9.2%
	Student	197	17.9%
	Taking care of household	45	4.1%
Financial status	Definitely satisfactory	162	14.8%
	Rather satisfactory	755	68.8%
	Rather unsatisfactory	155	14.1%
	Definitely unsatisfactory	26	2.4%
Experience with health care	Definitely good	56	5.1%
	Rather good	413	37.6%
	Neither good nor bad	385	35.1%
	Rather bad	207	18.9%
	Definitely bad	37	3.4%

a Combined categories (answers “d” and “e” in Q30).

b Combined categories (answers “f” and “h” in Q30).

c Combined categories (answers: “g” and “i” in Q30).

d Habilitation is the highest scientific degree in Poland awarded to scientists holding the PhD degree.

e Provided answers: medical and economic education.

f More than one answer was possible.

As for the two professional groups, which were supposed to be studied separately, 104 (9.5%) interviewees described themselves as only health care professionals while 343 interviewees (31.2%) as only science and research professionals, whereas 91 (8.3%) allocated themselves to both groups. Such large proportion of scientists and individuals associated with the scientific sector is consistent with the abovementioned results regarding the level of education or place of residence (major research centers are mostly located in large cities). In fact, 72.5% of science and research professionals lived in cities above 250,000 inhabitants while only 9.7% in villages and even less (9.4%) in cities up to 50,000 inhabitants. 91 respondents worked in both sectors (health care as well as science and research) which, given the similarities between these two sectors, was an expected outcome. Of note, both questions on profession also refer to the past, hence there is a possibility that someone from the group of 91 respondents worked in both sectors in the past, not necessarily at the time the questions were answered. We certainly hoped for a greater participation in the survey of physicians, who account for only 2.6% of all respondents. Such a small number may be due to the specificity of the profession (overload of duties, lack of time) and difficulties in reaching the medical staff in hospitals (multi-departmental facilities employing hundreds of people), but also the lack of interest in phage therapy as a form of treatment with unconfirmed effectiveness in standard clinical trials, which is also not reimbursed. However, according to The World Health Organization, there are 2.4 physicians per thousand inhabitants in Poland, i.e., still 10 times less than participated in our survey (44). The participation of individuals from the science and research sector, in turn, was high (39.5%), and even greater if we consider only Arm A (61.2% vs. 4.1% in Arm B). This can be explained by scientists' interest in phage therapy as a field of science that still needs investigation and the number of people more or less directly associated with the authors. More detailed characteristics of both professional groups are presented in Tables 2, 3.

**Table 2:** Core characteristics of health care sector (195 individuals).

**Characteristics**	**Categories**	**Number**	**Percentage within health care sector**	**Percentage of total^a^**
Sex	Female	147	75.4%	13.4%
	Male	48	24.6%	4.4%
Occupation	Physician	28	14.3%	2.6%
	Dentist	4	2.1%	0.4%
	Nurse/midwife	39	20.0%	3.6%
	Paramedic	5	2.6%	0.5%
	Rehabilitator/physiotherapist	11	5.6%	1.0%
	Laboratory diagnostician	13	6.7%	1.2%
	Pharmacist/pharmaceutical technician	19	9.7%	1.7%
	Other^b^	76	39.0%	6.9%
Medical specialization^c^	Anesthesiology and intensive care	2	7.1%	0.2%
	Surgery	2	7.1%	0.2%
	Internal medicine	8	28.6%	0.7%
	Occupational medicine	1	3.6%	0.1%
	Family medicine	1	3.6%	0.1%
	Neurology	1	3.6%	0.1%
	Oncology and palliative medicine	3	10.7%	0.3%
	Orthopedics	2	7.1%	0.2%
	Pediatrics	3	10.7%	0.1%
	Obstetrics and gynecology	1	3.6%	0.1%
	Medical rehabilitation	1	3.6%	0.1%
	Other	2	7.1%	0.2%
	No specialty	1	3.6%	0.1%

a percentage of the total study population (1,098 individuals).

b provided answers (among others): biotechnologist, microbiologist, researcher, academic teacher, lawyer, medical secretary.

c applies only to physicians.

**Table 3:** Core characteristics of science and research sector (434 individuals).

**Characteristics**	**Categories**	**Number**	**Percentage within science and research sector**	**Percentage of total^a^**
Sex	Female	303	69.8%	27.6%
	Male	131	30.2%	11.9%
Place of work	State university	113	26%	10.3%
	Private university	18	4.1%	1.6%
	Polish Academy of Sciences	246	56.7%	22.4%
	Research institute	9	2.1%	0.8%
	Other	48	11.1%	4.4%
Occupation	Researcher	175	40.3%	15.9%
	Research and teaching employee	64	14.7%	5.8%
	Teaching employee	27	6.2%	2.5%
	Research and development^b^	29	6.7%	2.6%
	Other	139	32%	12.7%
Years of experience	Up to 5 years	145	33.4%	13.2%
	6–10 years	77	17.7%	7.0%
	11–20 years	106	24.4%	9.7%
	21–30 years	64	14.7%	5.8%
	Over 30 years	42	9.7%	3.8%

a percentage of the total study population (1,098 individuals).

b activities performed outside of academic facility.

### 3.2 Analysis of quiz results and assessment of general knowledge about bacteriophages and phage therapy

Ultimately, 1,020 respondents (92.9%) agreed to participate in a test assessing General Knowledge about Bacteriophages (GKB) and their use in the treatment of infections caused by ABR bacteria. We have tried as much as possible to ensure that the questions in the quiz are not directly related to the information contained in the questionnaire. In other words, the answers required the knowledge that the respondent had before completing the questionnaire. Achieving this goal was not always possible. For instance, references to bacterial infections or the experimental nature of phage therapy were inevitable. On the other hand, questions directly related to the questionnaire were a kind of test of whether the respondents answered the questions with attention and understanding. A summary of all responses is presented in Table 4. A full list of questions and a set of answers (with the correct answers underlined) is available in Appendix 1.

**Table 4:** Summary of basic knowledge of phage therapy and research (question 59–70) and results of the reliability analysis of the GKB-12 survey items regarding General Knowledge about Bacteriophages and their use in the treatment of infections caused by ABR bacteria.

**Question^a^**	**Question subject**	**Correct answers**					**Percentage of selected answers; *I don't know***
		**Percentage of all answers**	**M** ^*^	**SD** ^*^	**r** ^*^	α^*^	
1 (59)	Bacteriophage origin	33.8%	4.68	2.65	0.479	0.749	23.2%
2 (60)	Prophage definition	46.6%	4.55	2.61	0.532	0.742	36.0%
3 (61)	Bacteriophage discovery (when)	18.4%	4.83	2.71	0.439	0.755	39.9%
4 (62)	Bacteriophage discovery (who)	26.6%	4.75	2.67	0.457	0.752	57.5%
5 (63)	Phage therapy centers	11.8%	4.90	2.76	0.381	0.761	61.0%
6 (64)	Phage therapy target	71.5%	4.31	2.73	0.302	0.769	7.2%
7 (65)	Infections treated at PTU	12.4%	4.74	2.72	0.338	0.765	60.3%
8 (66)	Form of phage therapy in Poland	75.8%	4.26	2.70	0.409	0.757	16.6%
9 (67)	Reimbursement of phage therapy costs	54.5%	4.47	2.72	0.277	0.773	38.5%
10 (68)	Manufacturing process	40.7%	4.61	2.62	0.512	0.745	39.3%
11 (69)	Bacteriophage biology	32.6%	4.69	2.70	0.353	0.763	33.7%
12 (70)	Bacteriophages vs. ABR bacteria	62.2%	4.39	2.65	0.462	0.751	28.4%

M^*^–mean if the item was removed; SD^*^–standard deviation if the item was removed; r^*^–correlation between individual questionnaire items and the total score (without a given item) if the item was removed; α^*^–Cronbach's α-value if the item was removed.

a original numbers of questions in questionnaire are given in brackets.

There were only 4 questions (out of 12) with more than half correct answers (Table 4). In Q8 we checked whether the respondent understood the form of phage therapy conducted in Poland (experimental or standard of care). As many as 75.8% of participants gave the correct answer to this question (*treatment conducted as a medical experiment*). References to the experimental form of phage therapy were found in many places in the questionnaire, which certainly contributed to such a high percentage of correct answers. By analogy, in Q6, we asked about group of microorganisms (infectious agents) that can be treated with bacteriophages. This question generated the second highest percentage of correct answers (71.5%). Such high percentage should not be surprising as the entire questionnaire was devoted to the main target of phage therapy i.e., bacterial infections. As previously stated, this is also evidence that the respondents filled out the questionnaire carefully and understood the questions they were asked. In fact, we can only assume what would be the result without completing the questionnaire in a first place. Two questions that caused the most problems, Q5 and Q7 with only 11.8% and 12.4% of correct answers respectively, focused on phage therapy facilities and their activity. Such outcome is consistent with results of our survey described above where 76.2% out of 1,098 examined respondents have never heard anything about PTU in Wrocław, even though this facility was mentioned earlier in our questionnaire. Because PTU activity is not known, respondents also could not indicate correctly which infections are treated at PTU in Wrocław (Q7) as they are not familiar with its activity.

Noteworthy, an active consent required to take part in the quiz unintentionally became a selection factor. We can assume that the people who agreed to participate in the quiz had some knowledge on the subject, otherwise they would not be so eager to do it. In fact, the study population was screened three times. The first step was to agree to participate in the survey. The second stage was the completion of the entire questionnaire, and the third stage was to agree to take part in the quiz. Hence, there is a possibility that the general knowledge of the population is at a lower level than indicated by this quiz.

The results of the quiz were used for the total assessment of knowledge, and on their basis, a scale of General Knowledge about Bacteriophages was built. The possible number of points for giving a correct answer ranged from 0 to 12. The proposed scale was validated by analyzing the reliability of the scale items (Table 4). The value of the Cronbach's α reliability index was estimated, which ranges from 0 to 1 (the higher the value, the greater the reliability of the scale). In the literature, it is assumed that a reliable scale is one for which α > 0.7 (45). Looking at the results of the summary reliability analysis, we can see that the internal consistency reliability for this sum was estimated at 0.775. The magnitude of the Cronbach's α for a summary scale consisting of only 12 items is good. This value can be interpreted in such a way that approximately 78% of the variability of the total score is the variability of the true score, i.e., the true variability among respondents due to the concept (bias) common to all items.

All questionnaire items correlated positively with the final grade, as evidenced by the values of correlation coefficients *r* ranging from 0.277 to 0.532, whereas average inter-item correlation coefficient was 0.225. The estimated internal consistency of the GKB 12 scale indicates that it can be considered a reliable tool for use in scientific research.

In the group of 1,020 respondents, the mean knowledge score was 5.01 points (SD = 2.90) that corresponds to a rather average level of knowledge. The distribution of results deviated significantly from the theoretical normal distribution (see Supplementary Figure 1), therefore non-parametric significance tests were used to compare average knowledge in subgroups of respondents differing in sociodemographic characteristics.

**Figure 1:**
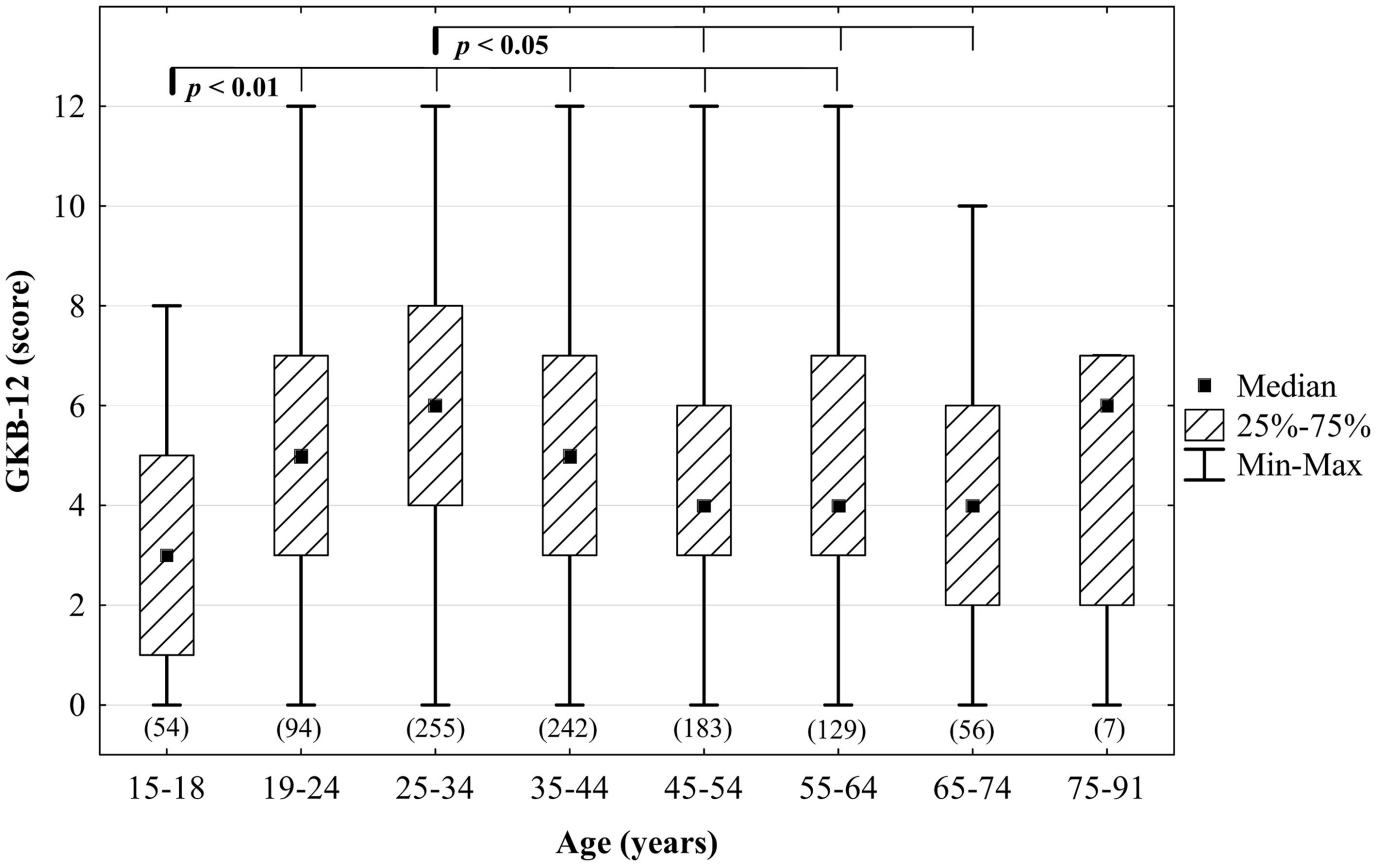
Assessment of knowledge about bacteriophages on the GKB-12 scale in different age groups. *Post-hoc* analysis was done using the Dunn's test (the sizes of the analyzed groups are given in brackets).

Detailed analysis of median GKB-12 score in different age groups was shown in Figure 1. The lowest score (median = 3 points) was observed in the group of respondents aged up to 18 years (*n* = 54; 5.3% of the total) whereas 255 respondents (25%) aged 25–34 had the greatest knowledge (median = 6 points). It was similar in the group of people over 74 years of age, but there were only 7 of them (0.7%) and therefore in *post-hoc* tests the differences compared to other age groups were statistically insignificant.

### 3.3 Perception of experimental therapies

Prior to analyzing the public's awareness and perception of phage therapy and research we wanted to have an initial idea about opinions on experimental treatment in general. Our efforts were motivated by two reasons. First of all, work on the survey began during the COVID-19 pandemic, which has brought the importance of medical research and development of experimental therapies to the forefront of public awareness. Secondly, phage therapy in the European Union, including Poland, is conducted solely on an experimental basis and the number of clinical trials involving bacteriophages is constantly growing (46). Our goal was to identify the potential connection between the overall perception of experimental therapies and phage treatment.

**Figure 2:**
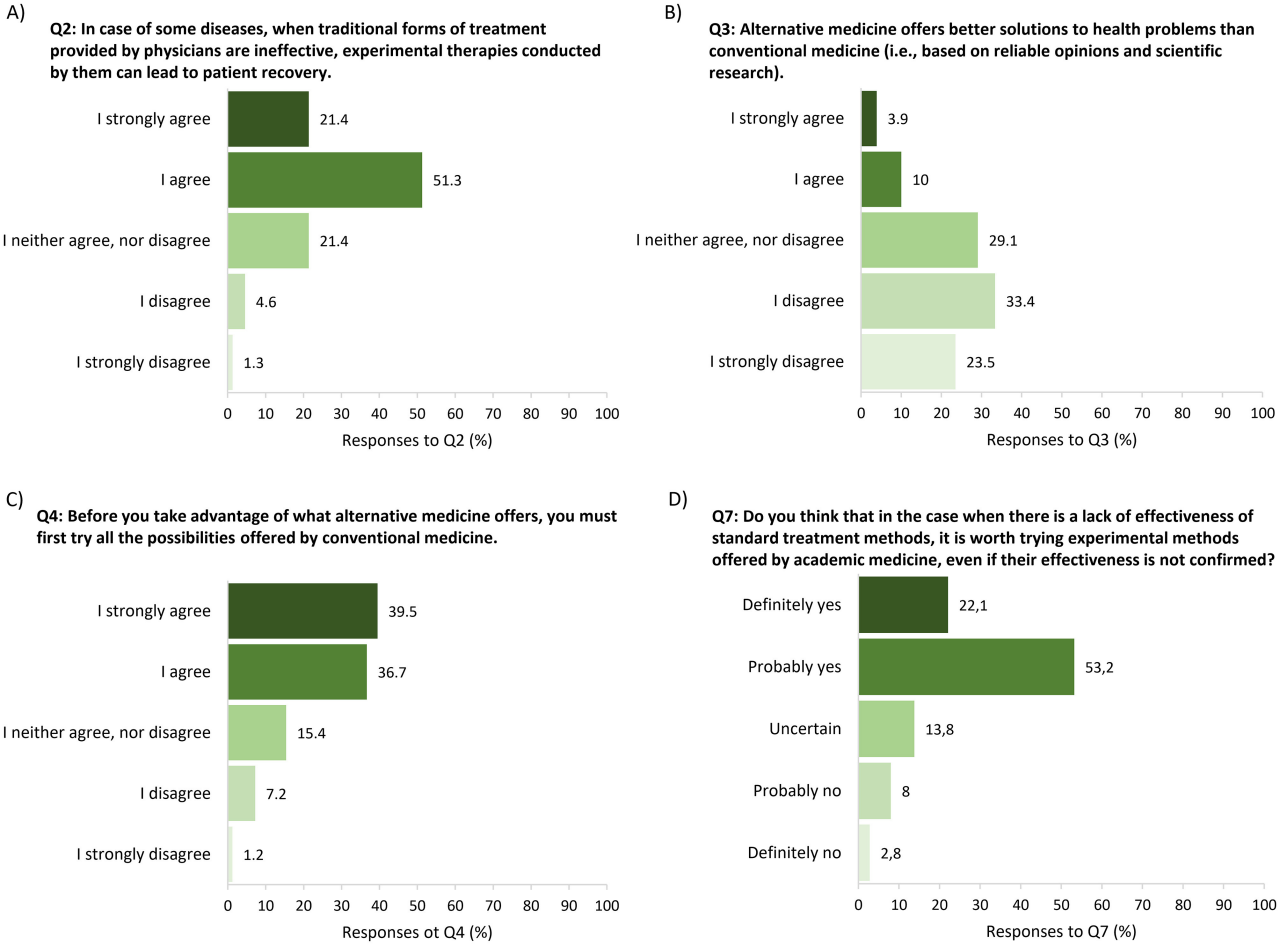
Responses to single-choice questions concerning perception of experimental therapies: **(A)** Q2; **(B)** Q3; **(C)** Q4; **(D)** Q7. Sample based on 1,098 responses.

Interesting outcomes regarding the perception of experimental therapies are shown in Figure 2. When asked about experimental therapies in general (Q2), respondents clearly acknowledged the need for such form of treatment (72.7%, Figure 2A). However, the question of whether experimental therapies prevail over the standard care (Q3) yielded quite the opposite response, i.e. the vast majority of respondents (86%, Figure 2B) did not agree with such a statement (the differences between Figures 2A, B are clearly visible). The distributions of answers to both questions are not uniform, which is confirmed by the result of the chi-square test (*p* < 0.001—see Supplementary Table 3). There is also a weak negative but statistically significant correlation (*r*_*S*_= −0.083, *p* < 0.05—see Supplementary Figure 2) between the ranks of answers to Q2 and Q3.

Those responses were somehow confirmed in the following Q4 emphasizing the priority of standard therapies over experimental ones, where over three-quarters of the respondents (76.2%) would try standard of care in the first place (Figure 2C). In a Q7 designed to check the credibility of the previously given answers, again, the majority (75.3%) confirmed the need for experimental therapies if there is no other treatment option (Figure 2D). Such prioritization of approved therapies is consistent with our PTU regulations, which require ineffective cycles of antibiotic therapy in each patient to be documented before they qualify for phage therapy. The result of the chi-square test indicated the existence of differences between the distributions of answers to Q4 and Q7 (*p* < 0.001—see Supplementary Table 4) and the ranks of answers to these questions correlated weekly but significantly from zero (*r*_*S*_= 0.073, *p* < 0.05—see Supplementary Figure 3). Notably, 79.1% of interviewees were vaccinated against SARS-Cov-2 and only 10% of them did not plan to take any vaccine against coronavirus (Q5). These numbers are consistent with the above-mentioned results indicating that during the COVID-19 pandemic vaccines against coronavirus were considered the most effective form of prevention in the absence of standard therapies and, thus, most of the respondents chose to be vaccinated. According to the Polish Ministry of Health, 22.8 million people were fully vaccinated against COVID-19 in Poland as of May 2023 (47). Such a number translates into 60% of the total population in Poland i.e., much lower when compared to the population investigated in this study (79.1%). According to the responders themselves, media hype around the COVID-19 pandemic (Q6) increased interest in experimental therapies in 27.8% (answers *definitely yes* or *probably yes*) whereas more than half of them (56.4%) provided opposite answers (*probably no* or *definitely no*; *p* < 0.001). Similar results were obtained in the group of respondents who were employees of the science and research sector. In more than half of the respondents (56.3%), events related to the COVID-19 pandemic did not influence their interest in experimental therapies, and only 10.4% answered this question *definitely yes* (*p* < 0.001).

### 3.4 Awareness and perception of antibiotic resistance

Nearly half of the respondents (48%) encountered (or someone from their family) the ineffectiveness of antibiotics, while exactly one quarter of respondents (25%) did not know or did not remember (*p* < 0.001; Q8). The analysis of responses to Q9 revealed an unexpectedly high percentage of respondents who had heard about the phenomenon of bacteria acquiring resistance to antibiotics (94.4%) which certainly does not correspond with results obtained by other authors (38, 48, 49). Given the high proportion of science and research as well as health care professionals among responders in Arm A we checked whether such percentage was also achieved in Arm B consisting of more random people, likely not associated with science. It turned out that the result was only slightly lower although the difference was statistically significant (96.6% for Arm A compared to 90.6% for Arm B; *p* < 0.001; Table 5). We decided to investigate this phenomenon further in order to identify a group with the highest and lowest awareness of antibiotic resistance. Despite some differences among particular groups, the overall percentage of awareness remains high with no group scoring <66.7% (Table 5). Most of the respondents heard or read about antibiotic resistance on TV, radio, in the press or on the Internet (62.5%), 36.7% had heard about it at school or university (but this result was twice as high among students and amounted to 66.5%), 16.3% were familiar with antibiotic resistance because they underwent infection with ABR bacteria and 3.5% were physicians who had encountered that problem at their work. Among the 14% of individuals who chose the last option in Q10 (answer *other*) the majority were scientists who dealt with antibiotic resistance on a professional level or people who had relatives or friends dealing with infection caused by ABR bacteria.

**Table 5:** Awareness of antibiotic resistance between both tested datasets and selected groups (1,098 individuals in total).

**Sample**	**Q9: Have you ever heard that sometimes bacteria that caused the disease can be resistant to antibiotics and that antibiotic therapy is then ineffective?**		***p*-value^e^**
	**Total number of respondents in a tested groups**	**Number of “yes” responses**	
Arm A	681	658 (96.6%)	**<0.001**
Arm B	417	378 (90.6%)	
Females	694	665 (95.8%)	**0.009**
Males	404	371 (91.8%)	
Inhabitants of villages	213	188 (88.3%)	**<0.001^f^**
Inhabitants of cities up to 50,000	165	151 (91.5%)	**<0.001^f^**
Inhabitants of cities 50,000–250,000	181	173 (95.6%)	**<0.001^f^**
Inhabitants of cities above 250,000	539	524 (97.2%)	**<0.001^f^**
Full-time employees	730	706 (96.7%)	**<0.001^f^**
Part-time employees^a^	75	75 (100%)	**0.028^f^**
Temporal, seasonal, commission work	93	88 (94.6%)	0.907^f^
Parental/maternity leave^b^	12	8 (66.7%)	**<0.001^f^**
Unemployed	33	29 (87.9%)	0.210^f^
Retired	101	97 (96%)	0.586^f^
Students	197	178 (90.4%)	**0.012^f^**
Taking care of household^b^	45	36 (80.0%)	**<0.001^f^**
Non-high school diploma^b^^,^^c^	147	112 (76.2%)	**<0.001^f^**
High school diploma^c^	230	218 (94.8%)	0.876^f^
Higher education^c^^,^^d^	718	704 (98.1%)	**<0.001^f^**
Only health care professionals^a^	104	103 (99.0%)	**<0.030^f^**
Only science and research professionals^a^	343	339 (98.8%)	**<0.001^f^**
Both health care and science/research professionals^a^	91	91 (100.0%)	**<0.015^f^**
Neither health care professionals nor science/research professionals	560	503 (89.8%)	**<0.001^f^**

a three groups with the highest awareness of antibiotic resistance.

b three groups with the lowest awareness of antibiotic resistance.

c combined categories.

d from a bachelor's degree onwards.

e statistically significant values (in Pearson chi-square) are highlighted in bold.

f calculated for a given category compared to the rest of responders.

**Figure 3:**
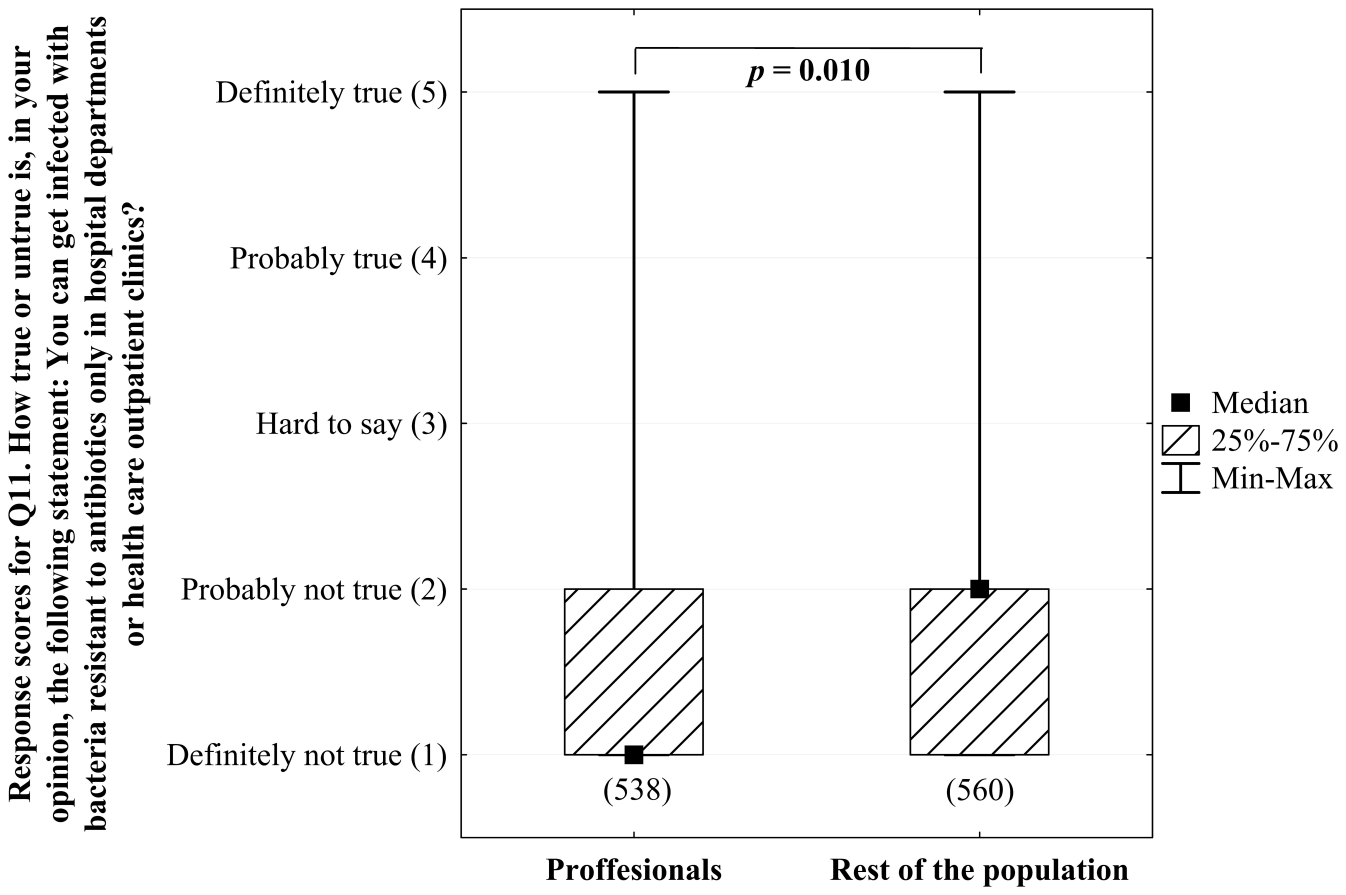
Comparison (in Mann-Whitney U test) of the awareness and perception of antibiotic resistance determined in Q11 (*How true or untrue is, in your opinion, the following statement: You can get infected with bacteria resistant to antibiotics only in hospital departments or healthcare outpatient clinics?*) between professionals (the employees of the health care and/or pharmaceutical sector) and the rest of responders (determined in Q33, the sizes of the analyzed groups are given in brackets below columns representing them).

When asked about potential source of infection with ABR bacteria (Q11) half of the respondents (49.9%) correctly identified that health care facilities are not the only source of such infections (answer *definitely not true*), however there were statistically significant differences between professionals and lay people (Figure 3). Concerns related to the possibility of getting infected with ABR bacteria (Q12) were expressed by 42.3% of responders in opposite to the 25.9% of those who did not afraid getting infected with such pathogens. We did not see any significant differences in this respect for health care professionals or science and research professionals vs. the rest of the population (Supplementary Table 5).

### 3.5 Awareness and perception of bacteriophages and phage therapy

**Figure 4:**
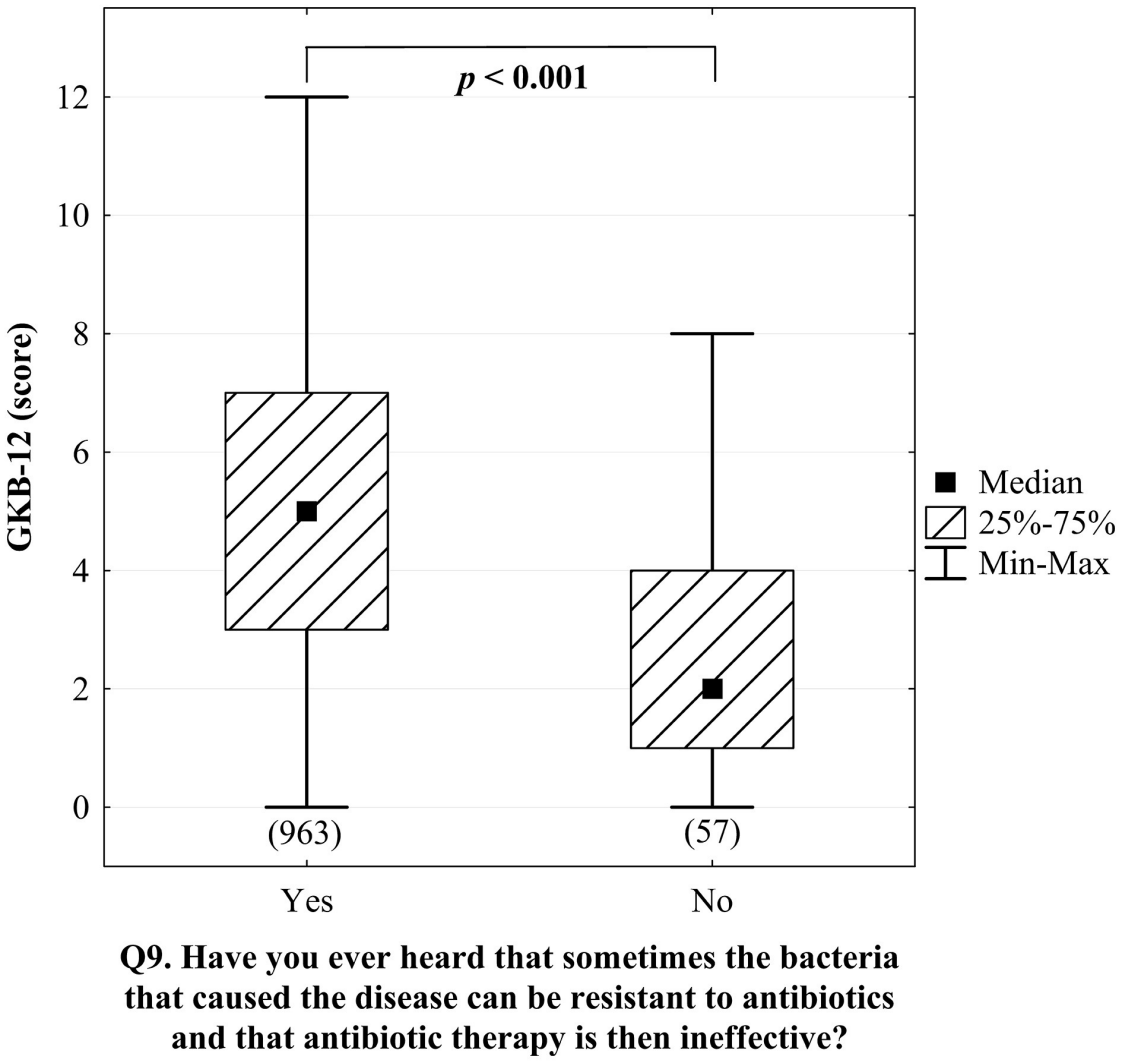
Assessment of knowledge about bacteriophages on the GKB-12 scale in groups of respondents differing in the answer to question Q9 (*Have you ever heard that sometimes the bacteria that caused the disease can be resistant to antibiotics and that antibiotic therapy is then ineffective?*) and the result of the Mann-Whitney significance test (the sizes of the analyzed groups are given in brackets).

**Figure 5:**
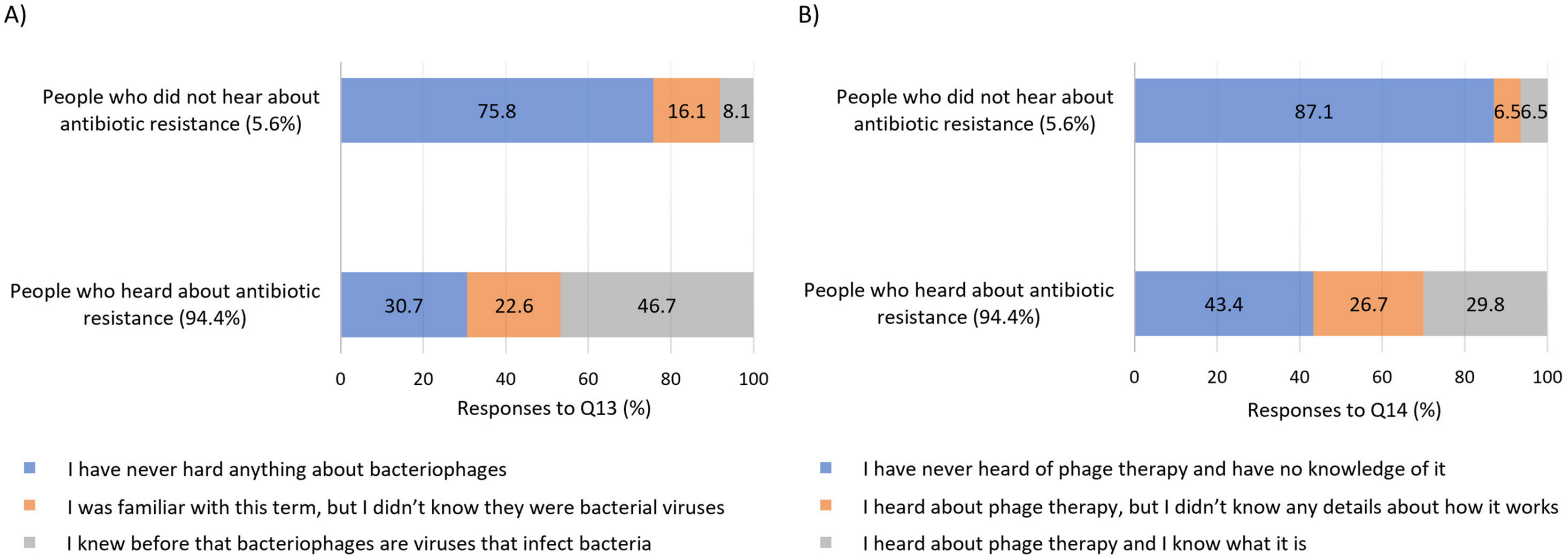
Single-choice questions concerning awareness of bacteriophages **(A)** and phage therapy **(B)** in connection with knowledge of antibiotic resistance. Sample based on 1,098 responses. Questions are as follows: Q13: *Bacteriophages (also called phages) are bacteria-specific viruses. They infect them, multiply in them, and after multiplying they can destroy them. Have you ever heard of bacteriophages before?* Q14: *Have you ever heard of phage therapy, which is based on the use of bacteriophages to treat infections caused, among others, by bacteria that have acquired resistance to various antibiotics?*

We started the analysis of the awareness of phage therapy by checking how much it corresponds with the awareness of antibiotic resistance of bacteria. It turned out that awareness of antibiotic resistance was closely associated with the score of general knowledge about bacteriophages and phage therapy (*p* < 0.001, Figure 4). Besides, between those who heard about antibiotic resistance one-third (30.7%) of respondents never heard anything about bacteriophages and 43.4% had never heard anything about phage therapy, whereas in a group of responders who never heard about antibiotic resistance those percentages were over twice higher (75.8% and 87.1% respectively) (Figure 5). Notably, awareness of antibiotic resistance (Q9; 94.4%) was twice as high as that of bacteriophages (Q13; 44.5%) and three times higher than that of phage therapy (Q14; 28.5%).

The two above-mentioned questions concerning consciousness about bacteriophages (Q13) and about phage therapy (Q14) were summarized by sex, age, place of residence, level of education, employment status, profession, financial status (Tables 6, 7 respectively). Considering the objectives of the survey, these two questions are crucial and give a cross-sectional picture of the public awareness of phage research. Interestingly, there were statistically significant differences in the answers given to these two questions with females demonstrating a higher level of knowledge and awareness of both bacteriophages and phage treatment. Both males and females recognized more the term *bacteriophage* (60.6% and 70.3% respectively) than *phage therapy* (it remained unknown for around half of males and females). We observed an analogous relationship among people with higher education, whose knowledge about bacteriophages and their nature (58.9% in Table 6) was much higher than that of the essence of phage therapy (37.7% in Table 7). Similarly, we found statistically significant outcomes depending on the place of residence, level of education, employment status and, as expected, between lay people and health care/science and research professionals (Tables 6, 7). In addition, there were statistically significant differences in knowledge rate among people with different financial status with the highest knowledge rate about bacteriophages (56.2%) and phage therapy (37%) attributed to people describing their material status as *definitely good*. We did not notice statistically significant differences in awareness of bacteriophages and phage therapy (Q13–14) depending on people's experience with health care (Q36) (data not shown). In the age category (data not shown), the lowest percentages of positive responses were noted among adolescents up to 18 years old (60.7% had never heard about anything about bacteriophages and 66.1% had never heard about phage therapy), and people above 60 years old (44.6% and 57.1% respectively). This was in line with the median GKB-12 scores (Figure 1) which were the lowest in adolescents. Interestingly, the correlation between all respondents' ratings on the GKB-12 scale and their answers to Q13 and Q14 was moderately positive (Spearman's *rho* was 0.553 and 0.571 respectively; *p* < 0.001—see Supplementary Figure 4). This may be due to the fact that the test questions were not easy, and the substantive knowledge about bacteriophages and phage therapy is much weaker than the awareness about them.

**Table 6:** Self-assessment by responders of their knowledge of bacteriophages (*n* = 1,098).

**Sample**	**Q13: Bacteriophages (also called phages) are bacteria-specific viruses. They infect them, multiply in them, and after multiplying they can destroy them. Have you ever heard of bacteriophages before?**			***p*-value^c^**
	**I have never heard anything about bacteriophages**	**I was familiar with this term, but I didn't know they were bacterial viruses**	**I knew before that bacteriophages are viruses that infect bacteria**	
Males	159 (39.4%)	103 (25.5%)	142 (35.1%)	**<0.001**
Females	206 (29.7%)	141 (20.3%)	347 (50.0%)	
Inhabitants of villages	109 (51.2%)	49 (23.0%)	55 (25.8%)	**<0.001^d^**
Inhabitants of cities up to 50,000	74 (44.8%)	31 (18.8%)	60 (36.4%)	**<0.001^d^**
Inhabitants of cities 50,000–250,000	74 (40.9%)	50 (27.6%)	57 (31.5%)	**<0.001^d^**
Inhabitants of cities above 250,000	108 (20.0%)	114 (21.2%)	317 (58.8%)	**<0.001^d^**
Full-time employees	195 (26.7%)	165 (22.6%)	369 (50.7%)	**<0.001^d^**
Part-time employees	26 (34.7%)	14 (18.7%)	35 (46.7%)	0.745^d^
Temporal, seasonal, commission work	21 (22.6%)	18 (19.4%)	54 (58.1%)	**0.018^d^**
Parental/maternity leave	8 (66.7%)	1 (8.3%)	3 (25.0%)	**0.046^d^**
Unemployed	18 (54.5%)	7 (21.2%)	8 (24.2%)	**0.020^d^**
Retired	56 (55.4%)	26 (25.7%)	19 (18.8%)	**<0.001^d^**
Students	63 (32.0%)	30 (15.2%)	104 (52.8%)	**0.011^d^**
Taking care of household	28 (62.2%)	10 (22.2%)	7 (15.6%)	**<0.001^d^**
Non-higher education^a^	228 (60.0%)	86 (22.6%)	66 (17.4%)	**<0.001**
Higher education^a^^,^^b^	137 (19.1%)	158 (22.0%)	423 (58.9%)	
Only health care professionals	25 (24.0%)	25 (24.0%)	54 (51.9%)	**<0.001^d^**
Only science and research professionals	29 (8.5%)	58 (16.9%)	256 (74.6%)	**<0.001^d^**
Both health care and science/research professionals	5 (5.5%)	14 (15.4%)	72 (79.1%)	**<0.001^d^**
Neither health care professionals nor science/research professionals	306 (54.6%)	147 (26.3%)	107 (19.1%)	**<0.001^d^**

a combined categories.

b from a bachelor's degree onwards.

c statistically significant values (in Pearson chi-square) are highlighted in bold.

d calculated for a given category compared to the rest of responders.

**Table 7:** Awareness of phage therapy in the study population (*n* = 1,098).

**Sample**	**Q14: Have you ever heard of phage therapy, which is based on the use of bacteriophages to treat infections caused, among others, by bacteria that have acquired resistance to various antibiotics?**			**p-value^c^**
	**I have never heard of phage therapy and have no knowledge of it**	**I have heard about phage therapy, but I did not know any details about how it works**	**I have heard about phage therapy and I know what it is**	
Males	204 (50.4%)	100 (24.8%)	100 (24.8%)	**0.043**
Females	300 (43.2%)	181 (26.1%)	213 (30.7%)	
Inhabitants of villages	123 (57.7%)	63 (29.6%)	27 (12.7%)	**<0.001^d^**
Inhabitants of cities up to 50,000	86 (52.1%)	47 (28.5%)	32 (19.4%)	**<0.001^d^**
Inhabitants of cities 50,000–250,000	98 (54.1%)	43 (23.8%)	40 (22.1%)	**<0.001^d^**
Inhabitants of cities above 250,000	197 (36.5%)	128 (23.7%)	214 (39.7%)	**<0.001^d^**
Full-time employees	302 (41.4%)	186 (25.5%)	242 (33.2%)	**<0.001^d^**
Part-time employees	29 (38.7%)	30 (40.0%)	16 (21.3%)	**0.012^d^**
Temporal, seasonal, commission work	40 (43.0%)	20 (21.5%)	33 (35.5%)	0.275^d^
Parental/maternity leave	9 (75.0%)	1 (8.3%)	2 (16.7%)	0.120^d^
Unemployed	19 (57.6%)	7 (21.2%)	7 (21.2%)	0.387^d^
Retired	64 (63.4%)	24 (23.8%)	13 (12.9%)	**<0.001^d^**
Students	77 (39.1%)	55 (27.9%)	65 (33.0%)	0.099^d^
Taking care of household	33 (73.3%)	8 (17.8%)	4 (8.9%)	**0.001^d^**
Non-higher education^a^	246 (64.7%)	92 (24.2%)	42 (11.1%)	**<0.001**
Higher education^a^^,^^b^	258 (35.9%)	189 (26.3%)	271 (37.7%)	
Only health care professionals	39 (37.5%)	40 (38.5%)	25 (24.0%)	**<0.001^d^**
Only science and research professionals	94 (27.4%)	78 (22.7%)	171 (49.9%)	**<0.001^d^**
Both health care and science/research professionals	12 (13.2%)	25 (27.5%)	54 (59.3%)	**<0.001^d^**
Neither health care professionals nor science/research professionals	359 (64.1%)	138 (24.6%)	63 (11.3%)	**<0.001^d^**

a combined categories.

b from a bachelor's degree onwards.

c statistically significant values (in Pearson chi-square) are highlighted in bold.

d calculated for a given category compared to the rest of responders.

Because respondents from Arm A could be more associated with us (e.g., by reaching them through e-mails or announcements on Institute's websites) than responders from Arm B (responses collected from an external source) additional analyses were conducted. We compared awareness of the existence of bacteriophages and phage therapy (Q13 and Q14) between lay people in Arm A and Arm B (Supplementary Tables 6, 7). Indeed, significantly more lay people from Arm A (*n* = 185) were familiar with term bacteriophages (64.9%) or phage therapy (51.4%) contrary to respondents (*n* = 375) from Arm B (35.7% and 28.3% respectively). Taking under consideration above differences and overrepresentation of health care as well as science and research professionals in our survey we can assume that the overall knowledge and awareness of bacteriophages and phage research in the Polish society is more similar to numbers attributed to Arm B.

**Figure 6:**
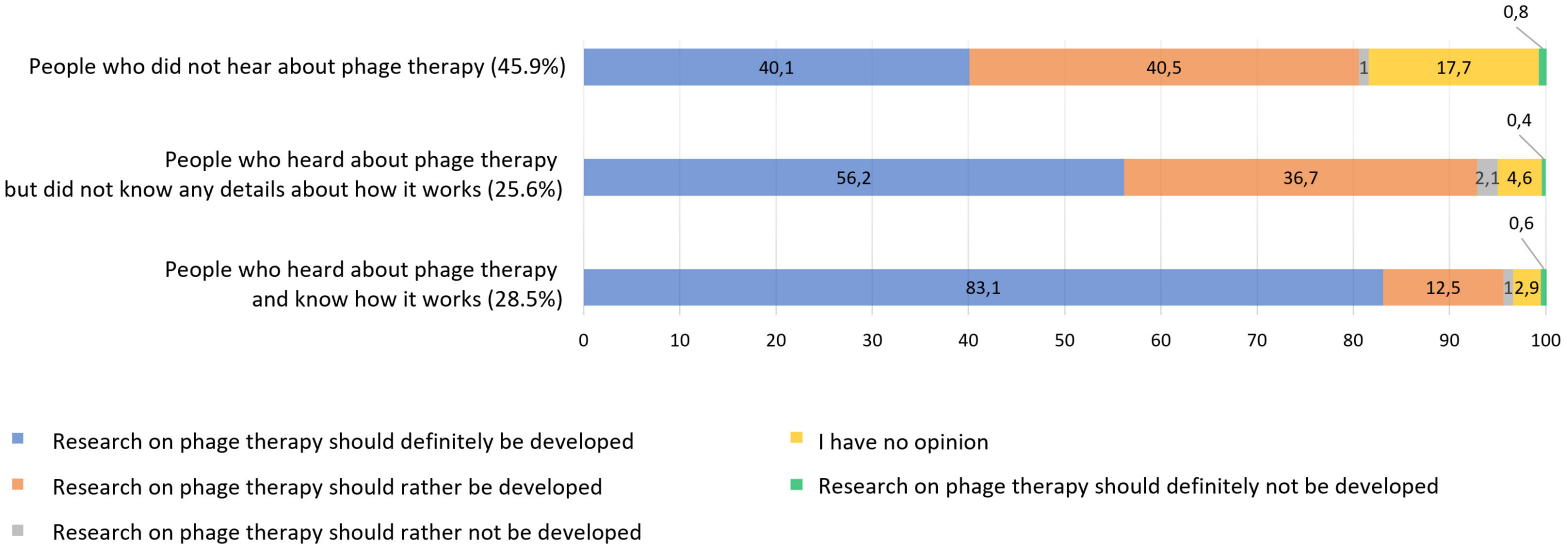
Comparison of awareness of phage therapy (Q14: *Have you ever heard of phage therapy, which is based on the use of bacteriophages to treat infections caused, among others, by bacteria that have acquired resistance to various antibiotics?*) and belief that it should be further developed (Q24: *Do you think that research on phage therapy in Poland should be further developed?*). Sample based on 1,098 responses. *p* < 0.001 in chi-square heterogeneity test.

The last analysis concerning the awareness of phage therapy involved question on whether research on phage therapy should be further developed in Poland (Q24). This idea got support from a very large percentage of all respondents (88.0%), but what is worth emphasizing also from lay people (83.2%). There was a clear correlation between awareness of phage therapy (Q14) and strong belief that research on phage treatment should be developed (Figure 6, Supplementary Figure 5). Twice as many people who have heard about phage therapy and know what it is (83.1%) support its development compared to the group (40.1%) who have never heard of it. This means that phage therapy has a positive connotation and high expectations are placed regarding its future among people who are familiar with it and it is in line with the general opinion that lack of knowledge leads to uncertainty and indifference.

Among respondents who had heard about phage treatment (Q15) the main source of knowledge was the Internet (28.2%), school/college (26.2%) and professional books and press (20.8%) while only 8% of respondents mentioned TV or radio, 9.5% family members or friends and 11.4% popular books and newspapers. The low percentage attributed to tv/radio and popular books/newspapers is an important indication that phage therapy has yet to break into the mainstream. When we analyzed the percentage of references to school/university depending on the age group (for details see Supplementary Table 8), it turned out that in the group of 15–18-year-olds it was 16.7%, and in the group of 19–24-year-olds it was as much as 44.2% whereas for responders older than 44 years it was below 16.5%. This means that rather a college education, and not elementary or secondary school likely contributes to this outcome, and phage therapy, as it has gained more attention in recent years, has been probably more often to be the subject of college lectures compared to the distant past.

In addition, we analyzed the knowledge of phage therapy among respondents who had heard of phage therapy (594 out of 1,098 respondents) as assessed by themselves (Q16). The largest group of respondents (17%) allocated themselves at the very bottom of the scale (*very little knowledge*; 1 point on the scale). Nearly three quarters of the respondents (69.4%) was in the first half of the scale (0–5) and only 4% rated their knowledge as high as possible (*a lot of knowledge*; 10 points on the scale). Science and research professionals (328 individuals) constituted a group most confident in their knowledge of phage therapy with the highest number of respondents among all tested groups allocating themselves between 6 and 10 points on the scale. Detailed results are shown in Supplementary Figure 6.

In line with these numbers indicating a rather low level of knowledge regarding phage treatment among the overall population, only 9.4% have visited the Database of Scientific Information Supporting Innovative Therapies (BINWIT) website (available at https://db.binwit.pl/en) so far. Nonetheless, most respondents (61.7%) expressed a willingness to deepen their knowledge by using the database on bacteriophages and phage therapy (Q25) and a readiness to visit the BINWIT website with only 12.5% of respondents not interested in such a project at all (Q26).

As for the experience with phage therapy, only 1.7% of respondents had been treated with phages, which is consistent with the experimental nature of this form of treatment, reserved only for participants who meet specific inclusion criteria. By analogy, only 3.6% had someone among relatives or friends who underwent phage therapy (Q17). 74.1% of respondents who heard anything about phage therapy found it to be *definitely safe* or *rather safe* (Q18) but the percentage of respondents who would agree to participate in a clinical trial on the use of bacteriophages (Q19) was lower (55.8%). We already showed that a lack of awareness of phage therapy develops a hesitancy toward research on it (Figure 6). That might also be a logical explanation as to why more people surveyed consider phage therapy to be safe than declare that they would participate in it.

Knowledge about the existence of PTU in Wrocław (Poland), the first ethically approved phage therapy center in the EU, is rather low. 76.2% out of 1,098 examined respondents had never heard anything about PTU. Such a result can be seen as quite surprising given the fact that half of the respondents were familiar with phage therapy. When we consider only the group who heard about phage therapy (54.1%), over half of them (58.4%) are still unaware of PTU's existence (Q20). Although PTU has been the subject of several articles and TV shows since its establishment in 2005 (16), we already demonstrated that only 8% of respondents had learned anything about phage therapy from TV and radio and only 11.4% from popular books and press. Clearly, this way of popularizing bacteriophages is not sufficient.

**Figure 7:**
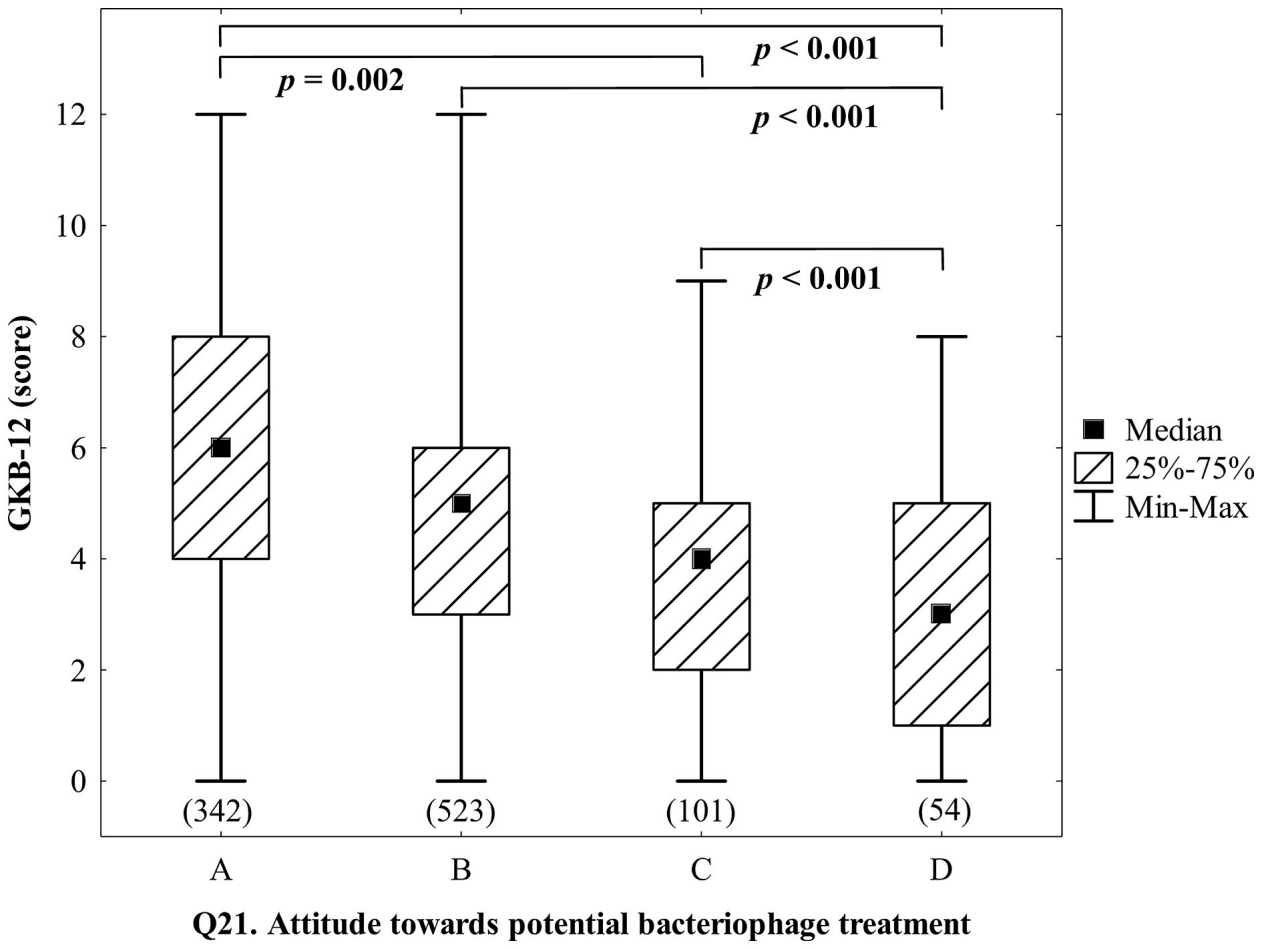
Attitude toward potential bacteriophage treatment (Q21: *If you fell ill with a disease caused by bacteria resistant to antibiotic therapy and antibiotics turned out to be ineffective in treatment, would you voluntarily undergo phage therapy conducted as part of a therapeutic experiment, if it was the only available method of treatment recommended by the attending physician, and its costs would have to be paid by you?*) and knowledge about bacteriophages according to the GKB-12 scale. Answers to Q21: (A) I would agree to such treatment; (B) I would agree to such treatment, but after obtaining additional positive opinions from other physicians; (C) I would rather not agree even if other physicians recommended such therapy to me; (D) I certainly would not agree to such treatment. Post-hoc analysis was done using the Dunn's test (the sizes of the analyzed groups are given in brackets).

Willingness to undergo phage treatment in the case of antibiotics failure was very high among all examined participants with only 5.8% of respondents stating that they would not agree to such a treatment while (84.4%) would agree to undergo phage treatment without hesitating or after obtaining additional opinions from other physicians. Notably, costs were not a deterrent factor although we did not specify the amounts to be paid which could potentially reduce interest further (Q21). Apparently, costs in general are of secondary importance when it comes to health with 84.4% of respondents (combined answers *a* and *b*) who would undergo such therapy with or without obtaining additional positive opinions from other physicians (Figure 7). On the other hand, it should be noted that the vast majority of respondents (83.6%) described their financial situation as *definitely satisfactory* or *rather satisfactory* (Table 1). Among the above-mentioned 5.8% respondents not willing to pay for phage treatment, half of them (54.4%) would undergo such treatment if they did not have to pay for it (Q22). By analogy to the willingness to incur costs of treatment, the necessity of personal visits to PTU in Wrocław would not constitute an obstacle for 59.6% of the interviewed respondents (Q23).

### 3.6 Perception of phage therapy and research by health care sector

Despite the long history of phage therapy in Poland, this form of treatment is still considered experimental by regulatory authorities and as such is not widely available or reimbursed. A relatively low number of physicians and dentists (*n* = 32) who took part in our survey seem to confirm this occurrence. A detailed characteristics of this group of interviewees can be found in Table 2.

Level of knowledge about phage therapy among physicians and dentists seems to be insufficient as well. Less than 10% of them described their knowledge as *completely sufficient*, half of them (50.0%) as *rather insufficient* and nearly one third (28.1%) as *definitely insufficient* (Q39). Even though 78.1% of physicians and dentists consider their knowledge insufficient, only 37.5% is ready to deepen their knowledge with additional 56.3% assuming such a possibility (Q40). In line with experimental status of phage therapy, Q42 revealed that only 9.4% of physicians used phages to treat their patients (6.3% more than once). The most noticeable finding is that a high percentage of physicians had never used phages in their practice despite dealing with patients who suffer from difficult to treat bacterial infections (65.6%). Even more surprising is the fact that 87.5% of examined physicians and dentists described antibiotic resistance of bacteria as a *very big threat* or *rather big threat* (Q43). This situation is characteristic not only for physicians but for the entire health care. When we asked all examined health care professionals whether have encountered the use of bacteriophages in their professional practice (Q41), most answers (85.6%) were negative (Supplementary Table 9).

Despite the low popularity of phage treatment in health care, 71.4% of physicians and dentists expressed their willingness to use phage therapy to treat their patients (Q45). Among the negative answers (*n* = 8), the lack of willingness to treat patients with phages was mostly attributed by responders (Q46) to little knowledge or experience in phage therapy (*n* = 3) or other not specified reasons (*n* = 3). Further, almost the same number of physicians assessed the possibility of implementing phage therapy in health care facilities where they work in two completely different ways i.e., as difficult and quite easy (43.8% and 40.6% respectively). Such discrepancy in Q44 may be the result of ignorance of what should be done to implement such therapy in health care facilities as we did not ask about it. The future of phage therapy among health care professionals is perceived rather positively with only 4.6% predicting a decline in its popularity (Table 8).

**Table 8:** Future of phage therapy as seen by 195 health care professionals (Q47).

**Answers to Q47**	**Frequency**	**Percentage**
Interest in phage therapy will deteriorate as a result of developing new antibiotics and other form of treatment for bacterial infections	9	4.6%
Phage therapy will be used in its current experimental form only as a “last resort” therapy	24	12.3%
Interest in phage therapy will increase, but it will remain a marginal form of antibacterial treatment	56	28.7%
Phage therapy will be used interchangeably with antibiotics	48	24.6%
In the future, phage therapy will marginalize other antibacterial therapies, including the use of antibiotics	13	6.7%
Hard to say	45	23.1%

### 3.7 Perception of phage therapy and research by science and research sector

Scientists constitute the largest group of respondents who agreed to take part in our survey (434 individuals, which is 39.5% of the entire tested population). Detailed characteristics of this group of interviewees can be found in Table 3. Interestingly, the vast majority of respondents from the science and research sector have never been involved in phage research and never plan to (80.2%) and at the same time 84.6% scientists find phage research interesting (Q51 and Q52 respectively). In Q53 we asked about the particularly interesting topic in phage research to confirm our observations that phage therapy seem to be the most popular aspect. The answers collected from 367 scientists who found phage research interesting confirmed our predictions i.e., that two most popular answers concern therapeutic connotations as therapeutic use of phages in the treatment of humans, animals and plants as well as manufacturing of phage preparations intended for therapy (Supplementary Table 10). However, only 14.1% expressed a strong belief that the most attractive is phage treatment in humans (Supplementary Figure 7A).

In line with current trends, most scientists recognized clinical trials as the most important factor contributing to further development of phage research in Poland. This answer was selected 361 times among 434 participating scientists (Q55). Consequently, nearly half of the examined scientists (49.1%) would increase public funds for research on the therapeutic use of bacteriophages, even at the expense of other research fields (Supplementary Figure 7B) which means they would be willing to sacrifice funds intended for other fields of science. However, there were statistically significant differences depending on the place of work. Surprisingly, researchers from institutes of the Polish Academy of Sciences were the least willing to increase funding for phages and constituted the largest group strongly opposed to such funding (Supplementary Table 11). They were significantly more critical compared to employees of state universities in terms of the need to increase public funds for research on the therapeutic use of bacteriophages (*p* = 0.047 in the Dunn's test when median values of responses were compared—see Supplementary Figure 8). In Q54, on factors that led to the initiation of phage research in the respondent's career, the most popular answer among 68 responders who declared their involvement in research related to bacteriophages was *joining a phage team* (66.2%) which may be a sign of the growing number of teams working in this field (which in fact is observed on a global scale). The comparison result had no significant relationship with the assessment of knowledge about bacteriophages. The level of scientists' knowledge assessed on the GKB-12 scale did not differ significantly, with the exception of employees of private universities (Supplementary Figure 9).

## 4 Discussion

One of the reasons for conducting surveys is to look for issues, perhaps hidden ones, that may arise in the future. Thus, a sociological study may be necessary to reveal an unexpected problem (50). As surveys on phage therapy are scarce, we can certainly think about looking ahead and identifying problems that may not have been identified before. In parallel to such a statement, we identified several trends and occurrences that were certainly surprising or unknown. Although clinical trials benefit everyone, perceptions of them are not always positive among the public, which is related to different beliefs, political and even religious views (51). There is no reason to believe that this is not the case with phage therapy, which is also seen as an experimental form of treatment. Additionally, our survey coincided with very interesting and unusual times of the global COVID-19 pandemic, which could have influenced the views of the respondents. According to The Center for Information and Study on Clinical Research Participation (CISCRP) in a study conducted on 10,010 Americans, the overall distrust in clinical trials increased three times during the pandemic (52). In addition, secondary bacterial infections placed an additional burden on COVID-19 patients which could be potentially associated with our survey concerning treating bacterial infections (13). However, based on the responses provided we did not notice an increase in interest in experimental therapies in more than half of the participants. The possible explanation is that, although the COVID-19 pandemic is still vivid in people's minds, we initiated our survey two and a half years after the start of the pandemic (June 2022), which may have contributed to information overload, including those about experimental therapies.

The urgency and severity of the COVID-19 pandemic have created a unique environment in which the demand for experimental treatments has skyrocketed. The public has witnessed the rapid development and deployment of therapies globally. As a result of this exposure and demand, the public perception of experimental therapies may have shifted. The public may be more open to the idea of participating in clinical trials and receiving experimental treatments, recognizing the potential benefits that these therapies may provide in the face of a serious illness. This shift may be particularly pronounced among individuals who have been directly affected by COVID-19. However, it is important to note that this change in perception may also be temporary. In addition, there are still important ethical and safety concerns to consider when it comes to experimental therapies, and it is essential to continue to prioritize safety and responsible research practices. Recently it has been emphasized that building awareness and understanding of clinical trials in Poland is of great importance (90% of subjects participating in clinical trials and 87.9% who did not participate in clinical trials desired more easily accessible information on clinical trials in newspapers, radio or TV) (51). Similarly, the majority of respondents in our study expressed a willingness to deepen their knowledge of phage therapy (61.7%). At the same time, TV/radio and newspapers constituted a marginal source of knowledge about phage treatment. The gap between expectations and reality is more than visible and the pertinency of sharing information about clinical trials and experimental therapies should not be ignored. Such differences are even more noticeable when we look into the popularity of PTU in Wrocław. More than half of the respondents who had heard about phage therapy, and more than 75% of the overall study population are not aware of PTU's existence, the only such center in Poland and one of only a few on a global scale. Without a doubt, much effort is needed to improve the knowledge and awareness of PTU serving as the only source of phage therapeutics in Poland. A desire for increased education on phage therapy and the need for increased awareness in this matter was recently emphasized by the authors conducting similar research in the UK (32). In addition, awareness of phage therapy is far behind that of antibiotic resistance which was very high (above 90%) among almost all tested groups with 62.5% of them gaining knowledge about it from the TV, radio, the Internet or newspapers. Notably, it has already been noted that a high level of media use (particularly TV, radio, newspaper, and social media) is associated with greater awareness of antimicrobial resistance (49) and phage treatment (28). The unexpectedly high level of awareness of antimicrobial resistance in our survey is hard to explain as it was not found in other sources (38, 48, 49, 53). However, other authors were more focused on antibiotic resistance in their research, asked more detailed questions, and created more opportunities for respondents to make mistakes. We only made mention of the problem (as it was not the focus), giving respondents the opportunity to select a positive answer without a deep understanding of the antibiotic resistance. It must be highlighted that a high awareness of antibiotics resistance among the tested population contradicted the low level of knowledge about associated risks (the highest proportion was observed in health care professionals where 17.9% described their concerns as *very afraid*). Such discrepancy confirms our assumptions that the respondents do not fully understand the phenomenon of antibiotic resistance and, again, it also indicates that these risks are not sufficiently highlighted in the media. In a most recent study by Alhur et al. the authors obtained similar results to ours based on 1,561 collected responses. While 75.72% of respondents had knowledge about antibiotic resistance and over 90% were aware of completing a course of antibiotic therapy, only slightly over 32% admitted that they followed this rule and did not complete the full course of prescribed antibiotics (54). The gap between the awareness of the problem and practice is noticeable and should be addressed by health care authorities in the future.

Among the obstacles we encountered while conducting the survey, the most important was access to respondents and the poor participation of physicians. Wide access to respondents thanks to the popularity of the Internet was rather fictional, as the Internet excluded many groups from the very beginning and is not a valuable source of solid respondents (36). Meyer et al. showed that web-based surveys in health care yielded the lowest response rate (46%) when compared to in-person surveys (76%), postal (65%) or even email ones (51%) (35). Moreover, the authors confirmed that health care professionals, including physicians, participated less often in postal and web surveys than patients due to lack of time, lack of potential benefits and excessive effort (of note, our questionnaire was characterized by a multi-level structure with expanded questions requiring thought and analysis). A similar problem was noted by Cho et al. who highlighted low and declining response rate to surveys involving health care providers based on 48 analyzed studies (55). The low response from physicians is also related to their low level of knowledge and experience about phage therapy which was pointed out by the physicians themselves (15, 28). Notably, we struggled not only with the physicians' low participation in the questionnaire, but also with their limited knowledge about phage therapy and even the willingness to deepen this knowledge, as we described above. Contrary to our results, a survey conducted in Scotland in 2023 (i.e., around the same time as our survey) highlighted significant awareness of phage therapy among clinicians, which was surprising for the authors themselves (56). However, such unexpected outcome may be the result of a survey conducted in hospitals that had experience with phage therapy. Similarly, the threats posed by antibiotic resistance were perceived to a greater extent by respondents from Scotland, probably due to recent Health Improvement Scotland recommendation for the use of phage therapy in patients with difficult to treat infections. These assumptions are confirmed by the fact that in Korea—a country where no activities related to phage therapy have been undertaken so far—only 10 out of 91 (11.0%) of the interviewed specialists rated their knowledge of phage therapy as high. However, respondents who identified themselves as well-informed on phage therapy were less concerned about its safety which emphasizes the importance of knowledge in building a positive climate around new forms of treatment (57).

Evaluating the population examined in our survey was challenging as it does not give a full picture of the opinions and views of Polish society. It is very difficult to collect a representative sample of the population in surveys devoted to topics at the intersection of science and medicine. Thus, only a specific group of people agreed to take part in the survey, which was the first selection threshold. Reaching by the respondents the end of the survey was another threshold as only individuals truly interested in the topic were most likely to be willing to complete our questionnaire. Last but not least, agreeing to participate in the optional quiz was the third threshold. Therefore, our study sample reflects all these elements (thresholds) of recruitment in terms of characteristics of respondents. We believe that all the positive numbers presented here would be smaller (and answers less encouraging) if the study population was more random. Nonetheless, given the lack of similar studies to date, our results constitute a good reference point for further research focusing on similar topic. They also provide a certain view on the perception of phage therapy in a society that is better educated, affluent and lives in large urban centers, which in the future could be comparable to other groups.

## 5 Conclusions

Overall, we found substantial differences in awareness of bacteriophages and phage therapy among respondents depending on the level of education (people with higher education vs. rest of the tested group) and occupational status (employees vs. unemployed or retired). Another unexpected finding is the lack of interest in phage therapy among physicians, who were not willing to complete our survey on the one hand, and those who did complete it were not interested in deepening their knowledge on the phage treatment. It should be expected that such physicians will not pass on information about phage therapy to their patients. Increasing the level of education in society and the amount of information in the media seems to be at least a partial remedy to these problems. As a matter of fact, we have shown a clear, directly proportional relationship between the level of awareness of bacteriophages and phage therapy and a positive attitude toward further development and funding of this type of treatment.

Looking at the increasing number of publications, start-ups and laboratories dealing with phages, along with the increasing number of clinical trials involving phages, the future looks rather promising. Irrespective of the final confirmation of the effectiveness of phage therapy, sociological research in this direction is yet to come.

## Data Availability

The raw data supporting the conclusions of this article will be made available by the authors, without undue reservation.
