# Supplementary material for: Perception of phage therapy and research across selected professional and social groups in Poland
Source: Front Public Health. 2025 Feb 11;13:1490737. doi: 10.3389/fpubh.2025.1490737 (PMC11884262; doi:10.3389/fpubh.2025.1490737)
Supplement: Supplementary file 1 [file Data_Sheet_1.docx]

Supplementary Material

# Supplementary Data

Appendix 1. The entire questionnaire with a full list of questions and answer options.

**Legend:** questions dedicated to a given group of responders are marked by colors as below:

| **questions intended for everyone**  **(including lay people)** | **questions intended for health care professionals**^a^ | **questions intended for science and research professionals**^b^ | **optional quiz for everyone completing the survey**^c^ |
| --- | --- | --- | --- |

a *based on the answer given in question 33;* ^b^ *based on the answer given in question 34;* ^c^ *based on the answer given in question 58*

| The survey questions will be answered by…  - 1. Woman   2. Man   In times of the COVID-19 coronavirus pandemic, much attention is paid to alternative or experimental forms of therapy. Please tell us to what extent you agree or disagree with the following statements: In case of some diseases, when traditional forms of treatment provided by physicians are ineffective, experimental therapies conducted by them can lead to patient recovery.  - 1. I strongly agree   2. I agree   3. I neither agree, nor disagree   4. I disagree   5. I strongly disagree  Alternative medicine offers better solutions to health problems than conventional medicine (i.e., based on reliable opinions and scientific research).  - 1. I strongly agree   2. I agree   3. I neither agree, nor disagree   4. I disagree   5. I strongly disagree  Before you take advantage of what alternative medicine offers, you must first try all the possibilities offered by conventional medicine.  - 1. I strongly agree   2. I agree   3. I neither agree, nor disagree   4. I disagree   5. I strongly disagree  Have you taken the SARS-CoV-2 coronavirus vaccine?  - 1. Yes   2. Not yet, but I plan to get vaccinated soon   3. No, but I am considering such a possibility depending on how the pandemic develops further   4. No, because I am not convinced about the efficacy or safety of the currently marketed vaccines   5. No, because I have medical contraindications for vaccination identified by a physician   6. I am not planning to take any vaccine against the coronavirus  Have the events related to the outbreak of the COVID-19 pandemic increased your interest in various experimental therapies offered by academic medicine?  - 1. Definitely yes   2. Probably yes   3. Uncertain   4. Probably no   5. Definitely no  Do you think that in the case when there is a lack of effectiveness of standard treatment methods, it is worth trying experimental methods offered by academic medicine, even if their effectiveness is not confirmed?  - 1. Definitely yes   2. Probably yes   3. Uncertain   4. Probably no   5. Definitely no  Antibiotics are drugs that are used to treat various bacterial diseases. Have you or anyone from your family or relatives ever been treated with antibiotics that proved to be ineffective?  - 1. Yes   2. No   3. I don’t know / I don’t remember  Have you ever heard that sometimes the bacteria that caused the disease can be resistant to antibiotics and that antibiotic therapy is then ineffective?  - 1. Yes   2. No  Under what circumstances have you come across the problem of diseases caused by bacteria resistant to antibiotic therapy? Please tick all possible options. [*conditional question, appears if question 9 is answered ‘a’*]   - 1. I heard or read about it on TV, radio, in the press or on the Internet   2. I heard about it during classes at school or university   3. I was infected with bacteria that are resistant to antibiotics   4. I am a physician and I see this problem in my patients at work   5. Other (what?)  How true or untrue is, in your opinion, the following statement: You can get infected with bacteria resistant to antibiotics only in hospital departments or healthcare outpatient clinics?  - 1. Definitely true   2. Probably true   3. Probably not true   4. Definitely not true   5. Hard to say  How worried are you about getting infected with antibiotic-resistant bacteria?  - 1. I am very afraid   2. I am afraid   3. I am neither afraid nor unafraid   4. I am unafraid   5. I am very unafraid  Bacteriophages (also called phages) are bacteria-specific viruses. They infect them, multiply in them, and after multiplying they can destroy them. Have you ever heard of bacteriophages before?  - 1. No, I have never heard anything about it   2. Yes, I was familiar with this term, but I didn't know they were bacterial viruses   3. Yes, I knew before that bacteriophages are viruses that infect bacteria  Have you ever heard of phage therapy, which is based on the use of bacteriophages to treat infections caused, among others, by bacteria that have acquired resistance to various antibiotics?  - 1. I heard about phage therapy and I know what it is   2. I heard about phage therapy, but I did not know any details about how it works and who it is intended for   3. I have never heard of phage therapy and have no knowledge of it  Where have you heard or read about phage therapy? Please select all that apply. [*conditional question, appears if question 14 is answered ‘a’ or ‘b’*]   - 1. in school/college   2. at work   3. in books and in the popular press   4. in the professional press, professional books   5. on the radio or TV   6. on the internet   7. from family members, friends, acquaintances   8. I don’t remember   9. in other sources (in which ones? Please enter your answer in the space provided)  How would you rate your knowledge of phage therapy on a scale of 1 to 10, where 1 means very little knowledge and 10 means a very high level of knowledge? [*conditional question, appears if question 14 is answered ‘a’ or ‘b’*]   - 1. 1 I have very little knowledge of phage therapy   2. 2   3. 3   4. 4   5. 5   6. 6   7. 7   8. 8   9. 9   10. 10 I have a lot of knowledge about phage therapy  Have you ever been treated with bacteriophages? Please select all that apply. [*conditional question, appears if question 14 is answered ‘a’ or ‘b’*]   - 1. Yes, I was treated with bacteriophages   2. One of my relatives and friends used them   3. I have never been treated with bacteriophages, nor have any of my relatives used this therapy  In your opinion, is phage therapy a safe or dangerous form of treatment for the patient? [*conditional question, appears if question 14 is answered ‘a’ or ‘b’*]   - 1. It is definitely safe   2. It is rather safe   3. It is rather not safe   4. It is definitely not safe   5. I don’t know  Clinical trials are conducted to discover or confirm the clinical or pharmacological effects of the study drug. They allow to determine the safety (e.g. the occurrence of adverse reactions) and the effectiveness of new drugs. Both healthy people (in phase 1 trials) and patients (in later phases) can participate in them. Thanks to this, patients also have the opportunity to use a new, not fully tested therapy, while accepting the associated risks. These studies are necessary for the manufacturer to obtain marketing authorization. Considering the above, how likely or not would you agree to participate in a clinical trial on the use of bacteriophages?  - 1. I would definitely agree   2. I would almost certainly agree   3. I would rather agree   4. I would rather not agreeI would almost certainly not agree   5. I would definitely not agree   6. Hard to say  So far, there are no results of clinical trials that would lead to the marketing authorization of therapeutic phage preparations and their use, e.g. as drugs available in pharmacies. Therefore, the Phage Therapy Unit in Wrocław conducts such treatment in the form of a therapeutic experiment, which is not a clinical trial and involves the use of new or only partially evaluated therapeutic methods in order to achieve a direct benefit to the patient's health. This treatment is provided for a fee (several thousand Polish zlotys) on an outpatient basis. A good result of therapy is observed in about 40% of treated patients with chronic (long-term course) bacterial infections resistant to antibiotic therapy. Have you heard of the Phage Therapy Unit in Wrocław before?  - 1. I have never heard   2. I heard, but I am not familiar with any details regarding its activity   3. I heard and I know that it conducts an experimental phage therapy  If you fell ill with a disease caused by bacteria resistant to antibiotic therapy and antibiotics turned out to be ineffective in treatment, would you voluntarily undergo phage therapy conducted as part of a therapeutic experiment, if it was the only available method of treatment recommended by the attending physician, and its costs would have to be paid by you?  - 1. I would agree to such treatment   2. I would agree to such treatment, but after obtaining additional positive opinions from other physicians   3. I would rather not agree even if other physicians recommended such therapy to me   4. I certainly would not agree to such treatment  If you fell ill with a disease caused by bacteria resistant to antibiotic therapy and antibiotics turned out to be ineffective in treatment, would you voluntarily undergo phage therapy conducted as part of a therapeutic experiment, if it was the only available method of treatment recommended by the attending physician, and you would not have to bear any associated costs? [*conditional question, appears if question 21 is answered ‘c’ or ‘d’*]   - 1. I would agree to such treatment   2. I would agree to such treatment, but after obtaining additional positive opinions from other physicians   3. I would rather not agree even if other physicians recommended such therapy to me   4. I certainly would not agree to such treatment  Would the requirement for on-site visits at the Phage Therapy Unit in Wrocław every few weeks be an obstacle for you in making the decision to start therapy, if you needed it and were determined to use it? [*conditional question, appears if question 21 or 22 are answered ‘a’ or ‘b’*]   - 1. It would be a significant obstacle   2. It would be an obstacle   3. It would hardly be an obstacle   4. It would not be an obstacle  Do you think that research on phage therapy in Poland should be further developed?  - 1. They should definitely be developed   2. They should rather be developed   3. They should rather not be developed   4. They definitely should not be developed   5. Uncertain  Would you be interested in using the database on bacteriophages and phage therapy?  - 1. Definitely yes   2. Probably yes   3. Uncertain   4. Probably no   5. Definitely no  Have you ever visited the website of the Database of Scientific Information Supporting Innovative Therapies (BINWIT)?  - 1. Yes, I visited, but only the home page   2. Yes, I visited and actively searched for information that I found interesting   3. I have not visited, but I intend to visit it   4. I have not visited but there is a chance I will visit it in the future   5. I have not visited and I am not interested in using this type of database   Now we have some questions about you personally. Your answers are very important to us. Thanks to them, we will be able to compare the answers of groups of people who differ from each other. What year were you born? Please enter 4 digits _ _ _ _Do you live permanently in Poland?  - 1. Yes   2. No  What kind of town do you live in? Is it:  - 1. Village   2. City up to 50,000 inhabitants   3. City between 50,000 and 250,000 inhabitants   4. City above 250,000 inhabitants  What is your education? We are asking about the highest level of education you have completed.  - 1. Unfinished elementary school   2. Completed elementary school   3. Completed secondary school   4. Completed basic vocational or related secondary vocational school after elementary school   5. Completed basic vocational school after secondary school   6. Completed high school without high school diploma   7. Completed high school with high school diploma   8. Completed technical school or vocational high school without high school diploma   9. Completed technical school or vocational high school with high school diploma   10. Completed post-high school education   11. I hold a bachelor's degree or an engineering degree   12. I hold a master's degree, a master's degree in engineering, a medical doctor's degree or another related degree   13. I hold a PhD degree   14. I hold a habilitated doctor's degree   15. I am a full professor   16. Other school or education  What other education do you have? We would like to remind you that we are asking about the highest level of education you have completed. [*conditional question, appears if question 30 is answered ‘p’*]  (Character limit: 300) What is your professional situation? Please select ALL answers that describe your professional situation.  - 1. I am a full-time employee   2. I am a part-time employee   3. I work temporarily, seasonally, on commission   4. I am on parental/maternity leave   5. I am unemployed and looking for a job   6. I'm retired, pensioner   7. I go to school, I am a student   8. I do not work/I take care of a household  Are you or have you been an employee of the healthcare or pharmaceutical sector (e.g. hospital, outpatient clinic, specialist outpatient clinic, health resort, doctor's office, physiotherapy office, pharmacy, pharmaceutical company, clinical research organization (CRO)?  - 1. Yes   2. No  Are you or have you been an employee of the science and research sector (e.g. Polish Academy of Sciences, research and development units, universities conducting research and development activities, an employee of the research department within a company, an academic teacher or a PhD student)?  - 1. Yes   2. No  How would you describe your financial situation?  - 1. Definitely satisfactory   2. Rather satisfactory   3. Rather unsatisfactory   4. Definitely unsatisfactory  What are your experiences as a patient with healthcare so far?  - 1. Definitely good   2. Rather good   3. Neither good nor bad   4. Rather bad   5. Definitely bad |
| --- |
| [*questions 37-47 below intended only for participants who answered ‘a’ in question 33*] Please select your occupation  - 1. Physician   2. Dentist   3. Nurse/midwife   4. Paramedic   5. Rehabilitator/physiotherapist   6. Laboratory diagnostician   7. Pharmacist/pharmaceutical technician   8. Other (please enter below)  Please specify the field of medicine you specialize in (in the case of several, please specify the one in which you are most professionally active) [*conditional question, appears if question 37 in answered ‘a’*]   - 1. Anesthesiology and intensive care   2. Balneology   3. Surgery   4. Internal medicine   5. Infectious diseases   6. Dermatology   7. Imaging diagnostics   8. Epidemiology   9. Geriatrics   10. Clinical immunology and transplantation medicine   11. Marine and tropical medicine   12. Occupational medicine   13. Emergency medicine   14. Family medicine   15. Medical microbiology   16. Neurosurgery   17. Neurology   18. Ophthalmology   19. Oncology and palliative medicine   20. Orthopedics   21. Otolaryngology   22. Pathology   23. Pediatrics   24. Obstetrics and gynecology   25. Psychiatry   26. Medical rehabilitation   27. Dentistry   28. Urology   29. Public health   30. Other   31. No specialization  Do you think that the knowledge you have about phage therapy is... [*conditional question, appears if question 37 in answered ‘a’ or ‘b’*]   - 1. Completely sufficient   2. Rather sufficient   3. Rather insufficient   4. Definitely insufficient  Would you like to expand your knowledge of phage therapy? [*conditional question, appears if question 37 in answered ‘a’ or ‘b’*]   - 1. Definitely yes   2. Probably yes   3. Probably no   4. Definitely no   5. Hard to say  Have you encountered the use of bacteriophages in your professional practice?  - 1. No, because in my professional practice I do not meet people who suffer from difficult to treat bacterial infections   2. No, although in my professional practice I meet people who suffer from difficult to treat bacterial infections   3. Yes, but it was an isolated case   4. Yes, more than once  Have you used bacteriophages in your medical practice? *[conditional question, appears if question 37 in answered ‘a’ or ‘b’]*   - 1. No, because I am not dealing with patients who suffer from difficult to treat bacterial infections   2. No, although I deal with patients who suffer from difficult to treat bacterial infections   3. Yes, but it was an isolated case   4. Yes, more than once  How big of a threat to patients, in your opinion, is the phenomenon of antibiotic resistance of bacteria? It is... [*conditional question, appears if question 37 in answered ‘a’ or ‘b’*]   - 1. a very big threat   2. a rather big threat   3. a rather minor threat   4. a very minor threat   5. not a threat at all  How do you assess the possibility of implementing phage therapy as an experimental form of treating patients in the facility where you work, if you had access to phage preparations? [*conditional question, appears if question 37 in answered ‘a’ or ‘b’*]   - 1. It is not possible at all in my opinion.   2. It would be difficult to implement   3. It would be quite easy to implement   4. It would be very easy to implement  Would you be willing to use experimental phage therapy to treat your patients? [*conditional question, appears if question 37 in answered ‘a’ or ‘b’ and question 42 is answered ‘b’*]   - 1. Yes   2. No  What is the reason why you would not be willing to use experimental phage therapy to treat your patients? (please select all that apply) [*conditional question, appears if question 37 in answered ‘a’ or ‘b’ and question 45 is answered ‘b’*]   - 1. Because reliable data on its effectiveness is not available   2. Because I am not satisfied with the effects of this therapy   3. Because obtaining the appropriate consent to implement this therapy is too complicated   4. Because I have no knowledge or experience in phage therapy   5. Because in my work I am not dealing with patients who would require such therapy   6. Another reason, which one?  What, in your opinion, will the future of phage therapy look like in the face of the growing antibiotic resistance of bacteria?  - 1. Interest in phage therapy will deteriorate as a result of developing new antibiotics and other form of treatment for bacterial infections   2. Phage therapy will be used in its current experimental form only as a "last resort" therapy   3. Interest in phage therapy will increase, but it will remain a marginal form of antibacterial treatment   4. Phage therapy will be used interchangeably with antibiotic therapy   5. In the future, phage therapy will marginalize other antibacterial therapies, including the use of antibiotics   6. Hard to say |
| [*questions 48-57 below intended only for participants who answered ‘a’ in question 34*] Please select the place of your work (if you have several places of work, please choose the one where you spend most of the time)  - 1. State university   2. Private university   3. Institute of the Polish Academy of Sciences   4. Research institute   5. Other  What is your current position?  - 1. I am a researcher   2. I am a research and teaching employee   3. I am a teaching employee   4. I do not work in an academic facility, but I conduct research and development activities   5. I hold a different position than mentioned above  How long have you been working or have you worked in the science and research sector?  - 1. Up to 5 years   2. 6 to 10 years   3. 11 to 20 years   4. 21 to 30 years   5. Over 30 years  Have you ever been involved in research related to bacteriophages?  - 1. I have never been involved in such research   2. I have not been involved so far, but I would like to be in the future   3. Yes, I was involved in such research in the past, but not anymore   4. Yes, I am currently involved in such research  Do you find research on bacteriophage interesting?  - 1. Yes   2. No   3. Hard to say  What topics related to bacteriophage research you find particularly interesting? (please select all that apply) [*conditional question, appears if question 52 is answered ‘a’*]   - 1. Phage biology (occurrence of phages, their isolation, biological properties)   2. Molecular biology of phages   3. Therapeutic use of phages in the treatment of humans, animals and plants   4. Manufacturing of phage preparations intended for therapy   5. Bacteriophage activity unrelated to their antibacterial properties   6. None of the above topics are of interest to me  What factors made you decide to start doing research on bacteriophages? (please select all that apply) [*conditional question, appears if question 51 is answered ‘c’ or ‘d’*]   - 1. Continuation of the research started while pursuing the bachelor's/master's/doctoral thesis   2. Promising prospects and dynamic development of this field   3. Joining a team that has already been involved in this field   4. Coincidence, such as the institutional research plans of the facility where I work  What, in your opinion, can have the greatest impact on the further development of research on bacteriophages in Poland? (please select all that apply)  - 1. Actions increasing access to experimental phage therapy, such as reimbursement of costs, simplification of procedures, increase in the number of centers conducting such therapy   2. Publication of the clinical trials results confirming the therapeutic effectiveness of bacteriophages   3. New discoveries related to non-antibacterial effects and applications of bacteriophages   4. Increased interest of the pharmaceutical industry in the therapeutic use of bacteriophages   5. In my opinion, the development of research on bacteriophages will continue independently of these factors   6. In my opinion, the development of research on bacteriophages soon will decrease   7. I have no opinion on that  To what extent do you agree or disagree with the statement that phage therapy in humans is the most attractive and promising field of research on bacteriophages?  - 1. I strongly agree   2. I agree   3. I neither agree, nor disagree   4. I disagree   5. I strongly disagree  In your opinion, should public funds be increased for research on the therapeutic use of bacteriophages, even at the expense of other research fields?  - 1. Definitely yes   2. Probably yes   3. Uncertain   4. Probably no   5. Definitely no |
| At the end of the survey, we would like to ask you to complete a short test consisting of 12 questions. These questions concern general knowledge about bacteriophages and their use in the treatment of bacterial infections that are resistant to antibiotics. Thanks to your and other people's answers, we will be able to assess the general level of knowledge on this subject. It shouldn't take you more than 5 minutes to complete the test. We would like to remind you that, like the rest of the survey you have completed so far, your answers to the test questions are completely anonymous. Do you agree to participate in the test?  - 1. Yes   2. No   [*questions 59-70 below intended only for participants who answered ‘a’ in question 58, i.e. who agreed to participate in the quiz; correct answers are underlined*] Bacteriophages are present in: (select all that apply)  - 1. seas and oceans   2. municipal and industrial wastewater   3. human and animal organisms   4. I don’t know  Prophage is:  - 1. bacteria susceptible to bacteriophage   2. bacteria resistant to bacteriophage   3. an inactive form of bacteriophage integrated into the bacterial genome   4. this term is used to describe any newly isolated bacteriophage   5. I don’t know  How many years ago were bacteriophages discovered?  - 1. less than 25 years ago   2. 25-50 years ago   3. 50-100 years ago   4. over 100 years ago   5. I don’t know  Bacteriophages were discovered by:  - 1. George Eliava   2. Felix d’Herell and Frederick Twort   3. Ludwik Hirszfeld   4. Louis Pasteur and Robert Koch   5. I don’t know  Which of the following centers conducting phage therapy is the oldest?  - 1. Queen Astrid Military Hospital in Brussels (Belgium)   2. Eliava Institute of Bacteriophages, Microbiology and Virology (IBMV) in Tbilisi (Georgia)   3. Phage Therapy Unit of the Medical Center of Hirszfeld Institute of Immunology and Experimental Therapy, Polish Academy of Sciences in Wrocław (Poland)   4. Center for Innovative Phage Applications and Therapeutics in San Diego (USA)   5. I don’t know  Phage therapy is currently used to treat infections caused by: (select all that apply)  - 1. viruses   2. bacteria   3. fungi   4. parasites   5. I don’t know  Which of the following diseases can be treated with bacteriophages in the Phage Therapy Unit in Wrocław? (select all that apply)  - 1. Lyme disease (caused by the spirochete *Borrelia burgdorferi*)   2. Chronic sinusitis caused by *Staphylococcus aureus*   3. Chronic gastritis caused by *Helicobacter pylori*   4. Chlamydia (genital infection caused by *Chlamydia trachomatis*)   5. I don’t know  The use of bacteriophages in the treatment of bacterial infections is currently considered in Poland as: (select all that apply):  - 1. standard method of treatment used in everyday medical practice   2. treatment conducted as a medical experiment   3. homeopathic form of treatment   4. taking dietary supplements   5. I don’t know  Are the costs of experimental phage therapy reimbursed?  - 1. Yes, they are fully reimbursed by the National Health Fund for all patients   2. Yes, they are fully reimbursed by the National Health Fund, but only for children   3. Yes, they are reimbursed by the National Health Fund, but only partially (up to 50% of the cost)   4. They are not reimbursed by the National Health Fund at all   5. I don’t know  Do you need bacteria in the manufacturing process of bacteriophage preparations?  - 1. No, they are not needed at all   2. Yes, they are needed at the initial stage of the manufacturing process for phage propagation, next they are discarded   3. Yes, they are needed at every stage of the manufacturing process and along with bacteriophages they can be found in the final phage preparation   4. Yes, bacteria are added to the final bacteriophage preparation   5. I don’t know  Which of the following statements about bacteriophages are true?  - 1. Mechanism of action of bacteriophages is the same as that of antibiotics   2. Bacteria do not acquire resistance to bacteriophages   3. Bacteriophages are able to propagate at the site of infection   4. Yes, they are needed at every stage of the manufacturing process and along with bacteriophages they can be found in the final phage preparation   5. Due to the broad spectrum of antibacterial activity, bacteriophages often cause disturbances in the intestinal flora   6. I don’t know  Can bacteriophages destroy (lyse) antibiotic-resistant bacteria?  - 1. Yes, such bacteriophage feature has been confirmed in scientific research   2. No, because bacteria that are resistant to antibiotics are also resistant to bacteriophages   3. It is not known because no one has investigated it yet   4. Previous scientific research does not confirm this   5. I don’t know |

Appendix 2. List of organizations and institutions in Poland informed about the survey with a request to distribute it among their employees.

**Legend:** groups of organizations and institutions are marked by colors as below:

| **patient’s associations** | **institutes of the Polish Academy of Sciences** | **health care entities** | **private and public universities** |
| --- | --- | --- | --- |

| **Name of the organization/institution** | **Website** | **Mailing date** |
| --- | --- | --- |
| - Federation of Polish Patients - Association *The Patient Is Most Important* - Polish Coeliac Society - Polish Society for Combating Cystic Fibrosis - Polish Coalition of Oncological Patients - Polish Stoma Association *Pol-ilko* - Association of People with Incontinence *UroConti* - Polish Multiple Sclerosis Society - National Association of Patients with Heart and Vascular Diseases   *EcoSerce*   - Polish Diabetes Association - Institute of Patient Rights and Health Education | - [http://federacjapp.pl](http://federacjapp.pl/) - [http://www.spjn.pl](http://www.spjn.pl/) - https://celiakia.pl - https://ptwm.org.pl - [https://www.pkpo.pl](http://www.pkpo.pl/) - [http://www.polilko.pl](http://www.polilko.pl/) - https://uroconti.pl - https://ptsr.org.pl - [http://ecoserce.pl](http://ecoserce.pl/) - https://diabetyk.org.pl - https://ippez.pl | October 25, 2022 |
| - Institut of Archeology and Ethnology - Institute of Literary Research - Institute of Philosophy and Sociology - Tadeusz Manteuffel Institute of History - Institute for the History of Science - Institute of Polish Language - Institute of Mediterranean and Oriental Cultures - Institute of Economics - Institute of Law Studies - Institute of Psychology - Institute of Rural and Agricultural Development - Institute of Slavic Studies - Institute of Political Studies - Institute of Art - European Regional Centre for Ecohydrology - Institute of Agrophysics - Institute of Biochemistry and Biophysics - Nencki Institute of Experimental Biology - Mammal Research Institute - Szafer Institute of Botany - Institute of Bioorganic Chemistry - Institute of Dendrology - Kielanowski Institute of Animal Physiology and Nutrition - Franciszek Górski Institute of Plant Physiology - Institute of Genetics and Animal Biotechnology - Institute of Plant Genetics - Institute of Nature Conservation - Institute of Paleobiology - W. Stefański Institute of Parasitology - Institute of Animal Reproduction and Food Research - Institute of Systematics and Evolution of Animals - Museum and Institute of Zoology - Nicolaus Copernicus Astronomical Center - Space Research Centre - Centre of Molecular and Macromolecular Studies - Center for Theoretical Physics - Centre of Polymer and Carbon Materials - Institute of Physical Chemistry - Institute of Organic Chemistry - Institute of Physics - Henryk Niewodniczański Institute of Nuclear Physics - Institute of Molecular Physics - Institute of Geophysics - Jerzy Haber Institute of Catalysis and Surface Chemistry - Institute of Mathematics - Institute of Geological Sciences - Institute of Low Temperature and Structural Research - Institute of Oceanology - Institute of High Pressure Physics - Systems Research Institute - Nalecz Institute of Biocybernetics and Biomedical Engineering - Institute of Hydroengineering - Stanisław Leszczycki Institute of Geography and Spatial Organization - Mineral and Energy Economy Research Institute - Institute of Theoretical and Applied Informatics - Institute of Chemical Engineering - Institute of Fluid-Flow Machinery - Strata Mechanics Research Institute - Institute of Metallurgy and Materials Science - Institute of Computer Science - Institute of Environmental Engineering - Institute of Fundamental Technological Research - Institute of Medical Biology - Maj Institute of Pharmacology - Institute of Human Genetics - Mossakowski Medical Research Institute - Hirszfeld Institute of Immunology and Experimental Therapy | - [http://iaepan.edu.pl](http://iaepan.edu.pl/) - https://ibl.waw.pl - https://ifispan.pl - https://ihpan.edu.pl - [https://www.ihnpan.pl](http://www.ihnpan.pl/) - https://ijp.pan.pl - [http://www.iksiopan.pl](http://www.iksiopan.pl/) - https://inepan.pl - https://inp.pan.pl - https://psych.pan.pl - [https://www.irwirpan.waw.pl](http://www.irwirpan.waw.pl/) - https://ispan.waw.pl - https://isppan.waw.pl - [http://www.ispan.pl](http://www.ispan.pl/) - [https://www.erceunescolodz.org](http://www.erceunescolodz.org/) - [https://www.ipan.lublin.pl](http://www.ipan.lublin.pl/) - https://ibb.edu.pl - https://nencki.edu.pl - https://ibs.bialowieza.pl - [https://www.botany.pl](http://www.botany.pl/) - [https://www.ibch.poznan.pl](http://www.ibch.poznan.pl/) - [https://www.idpan.poznan.pl](http://www.idpan.poznan.pl/) - [https://www.ifzz.pl](http://www.ifzz.pl/) - https://ifr-pan.edu.pl - [https://www.igbzpan.pl](http://www.igbzpan.pl/) - [http://www.igr.poznan.pl](http://www.igr.poznan.pl/) - [https://www.iop.krakow.pl](http://www.iop.krakow.pl/) - [https://www.paleo.pan.pl](http://www.paleo.pan.pl/) - [http://ipar.pan.pl](http://ipar.pan.pl/) - https://pan.olsztyn.pl - [http://www.isez.pan.krakow.pl](http://www.isez.pan.krakow.pl/) - https://miiz.waw.pl - [https://www.camk.edu.pl](http://www.camk.edu.pl/) - https://cbkpan.pl - [https://www.cbmm.lodz.pl](http://www.cbmm.lodz.pl/) - [https://www.cft.edu.pl](http://www.cft.edu.pl/) - https://cmpw-pan.edu.pl - https://ichf.edu.pl - [https://www.icho.edu.pl](http://www.icho.edu.pl/) - [https://www.ifpan.edu.pl](http://www.ifpan.edu.pl/) - [https://www.ifj.edu.pl](http://www.ifj.edu.pl/) - https://www.ifmpan.poznan.pl - [https://www.igf.edu.pl](http://www.igf.edu.pl/) - https://ikifp.edu.pl - [https://www.impan.pl](http://www.impan.pl/) - [https://www.ing.pan.pl](http://www.ing.pan.pl/) - [https://www.intibs.pl](http://www.intibs.pl/) - [https://www.iopan.gda.pl](http://www.iopan.gda.pl/) - [https://www.unipress.waw.pl](http://www.unipress.waw.pl/) - [https://www.ibspan.waw.pl](http://www.ibspan.waw.pl/) - [https://www.ibib.waw.pl](http://www.ibib.waw.pl/) - [http://www.ibwpan.gda.pl](http://www.ibwpan.gda.pl/) - [https://www.igipz.pan.pl](http://www.igipz.pan.pl/) - https://min-pan.krakow.pl - [https://www.iitis.pl](http://www.iitis.pl/) - [https://www.iich.gliwice.pl](http://www.iich.gliwice.pl/) - [https://www.imp.gda.pl](http://www.imp.gda.pl/) - https://imgpan.pl - [https://www.imim.pl](http://www.imim.pl/) - [https://www.ipipan.waw.pl](http://www.ipipan.waw.pl/) - [http://www.ipis.pan.pl](http://www.ipis.pan.pl/) - [https://www.ippt.pan.pl](http://www.ippt.pan.pl/) - [http://ibmpan.pl](http://ibmpan.pl/) - [http://if-pan.krakow.pl](http://if-pan.krakow.pl/) - https://igcz.poznan.pl - [https://www.imdik.pan.pl](http://www.imdik.pan.pl/) - https://hirszfeld.pl | December 8, 2022 |
| - Jan Biziel University Hospital No. 2 in Bydgoszcz - N. Copernicus Provincial Multidisciplinary Centre of Oncology and Traumatology in Łódź - J. Dietl Specialist Hospital in Kraków - S. Żeromski Specialist Hospital in Kraków - Institute of the Polish Mother's Memorial Hospital - Center for Invasive Cardiology *IKARDIA* in Nałęczów - Maria Skłodowska-Curie National Research institute of Oncology in Warsaw - L. Rydygier Specialist Hospital in Kraków - Silesian Center for Heart Diseases in Zabrze - J. Korczak Provincial Specialist Hospital in Słupsk - Central Clinical Hospital of the Ministry of Interior and Administration in Warsaw - University Children's Hospital in Lublin - University Teaching Hospital in Białystok - St. John of Dukla Oncology Centre of the Lublin Region - K. Marcinkowski University Hospital in Zielona Góra | - [https://www.biziel.umk.pl](http://www.biziel.umk.pl/) - [https://www.kopernik.lodz.pl](http://www.kopernik.lodz.pl/) - [http://szpitaldietla.pl](http://szpitaldietla.pl/) - https://zeromski-szpital.pl - [https://www.iczmp.edu.pl](http://www.iczmp.edu.pl/) - [http://szpital.ikardia.pl](http://szpital.ikardia.pl/) - [https://www.nio.gov.pl](http://www.nio.gov.pl/) - [https://www.szpitalrydygier.pl](http://www.szpitalrydygier.pl/) - [https://www.sccs.pl](http://www.sccs.pl/) - https://szpital.slupsk.pl - [https://www.gov.pl/web/cskmswi](http://www.gov.pl/web/cskmswi) a - [https://www.uszd.lublin.pl](http://www.uszd.lublin.pl/) - https://uskwb.pl - https://cozl.eu - https://szpital.zgora.pl | February 14, 2023 |
| - L. Zemnhof University Children’s Teaching Hospital in Białystok - A. Jurasz University Hospital No. 1 in Bydgoszcz - University Clinical Center in Gdańsk - A. Mielęcki Independent Public Teaching Hospital of the Medical University of Silesia in Katowice - Upper Silesian Child Health Center in Katowice - S. Szyszko Independent Public Teaching Hospital No. 1 of the Medical University of Silesia in Katowice - N. Barlicki University Teaching Hospital in Łódź - Central Teaching Hospital of the Medical University of Łódź - University Teaching Hospital in Poznań - Wiktor Dega Teaching Hospital of Orthopedics and Rehabilitation of the Poznań University of Medical Sciences - T. Sokołowski Independent Public Teaching Hospital No. 1 of the Pomeranian Medical University in Szczecin - University Clinical Center of the Medical University of Warsaw - Military Institute of Medicine – National Research Institute - University Teaching Hospital in Opole - 4^th^ Military Clinical Hospital in Wrocław - St. Queen Jadwiga Clinical Provincial Hospital No. 2 in Rzeszów - Military Medical Academy Teaching Hospital – Central Veterans' Hospital in Łódź - 1^st^ Military Teaching Hospital with Polyclinic of Independent Public Health Care Unit in Lublin - Gruca Orthopaedic and Trauma Teaching Hospital - F. Chopin University Teaching Hospital in Rzeszów - 5^th^ Military Clinical Hospital with Polyclinic of Independent Public Health Care Center in Kraków - J. Babiński Teaching Hospital in Kraków - Independent Public Clinical Hospital No. 2 of the Pomeranian Medical University in Szczecin - 10th Military Clinical Hospital with Polyclinic of Independent PublicHealth Care Center in Bydgoszcz - University Teaching Hospital in Wrocław - Department of Infectious and Tropical Diseases, Jagiellonian University Medical College - University Teaching Hospital in Olsztyn - Princess Anna Mazowiecka Clinical Hospital in Warsaw | - https://udsk.pl - https://jurasza.umk.pl - https://uck.pl - https://spskm.katowice.pl - https://gczd.katowice.pl - [https://www.szpital.zabrze.pl](http://www.szpital.zabrze.pl/) - [https://www.barlicki.pl](http://www.barlicki.pl/) - [http://www.csk.umed.pl](http://www.csk.umed.pl/) - [https://www.usk.poznan.pl](http://www.usk.poznan.pl/) - https://orsk.pl - [https://www.spsk1.szn.pl](http://www.spsk1.szn.pl/) - https://uckwum.pl - https://wim.mil.pl - [https://www.usk.opole.pl](http://www.usk.opole.pl/) - [https://www.4wsk.pl](http://www.4wsk.pl/) - https://szpital2.rzeszow.pl - https://skwam.lodz.pl - [http://1wszk.pl](http://1wszk.pl/) - [http://spskgrucy.pl](http://spskgrucy.pl/) - https://szpital.rzeszow.pl - https://5wszk.com.pl - https://nowa.babinski.pl - https://spsk2-szczecin.pl - [https://www.10wsk.mil.pl](http://www.10wsk.mil.pl/) - [http://www.usk.wroc.pl](http://www.usk.wroc.pl/) - https://zakazna.wl.cm.uj.edu.pl - [https://www.szpital.uwm.edu.pl](http://www.szpital.uwm.edu.pl/) - [https://www.szpitalkarowa.pl](http://www.szpitalkarowa.pl/) | March 9, 2023 |
| - University of Opole (Faculty of Medicine) - Medical University of Białystok - Medical University of Gdańsk - Medical University of Silesia - Medical University of Lublin - Medical University of Łódź - Poznań University of Medical Sciences - Pomeranian Medical University - Medical University of Warsaw - Wrocław Medical University - Collegium Medicum of the University of Zielona Góra - Ludwik Rydygier Collegium Medicum in Bydgoszcz of the Nicolaus Copernicus University in Toruń | - [http://im.wmnoz.uni.opole.pl](http://im.wmnoz.uni.opole.pl/) - [https://www.umb.edu.pl](http://www.umb.edu.pl/) - https://gumed.edu.pl - https://sum.edu.pl - [https://www.umlub.pl](http://www.umlub.pl/) - https://umed.pl - [https://www.ump.edu.pl](http://www.ump.edu.pl/) - [https://www.pum.edu.pl](http://www.pum.edu.pl/) - [https://www.wum.edu.pl](http://www.wum.edu.pl/) - [https://www.umw.edu.pl/pl](http://www.umw.edu.pl/pl) - https://cm.uz.zgora.pl - [https://www.cm.umk.pl](http://www.cm.umk.pl/) | March 16, 2023 |
| - AGH University of Kraków - Kraków University of Economics - Kraków University of Technology - Pedagogical University of Kraków - University of Physical Education in Kraków - Academy of Fine Arts in Kraków - Krzysztof Penderecki Academy of Music in Kraków - Jagiellonian University - School of Management and Banking in Kraków - Pontifical University of John Paul II in Kraków - Tischner European University - Cardinal Stefan Wyszyński University in Warsaw - Warsaw University of Life Sciences - Warsaw University of Technology - Military University of Technology in Warsaw - Academy of Fine Arts in Warsaw - Warsaw School of Economics - Chopin University of Music - Józef Piłsudski University of Physical Education in Warsaw - University of Warsaw - Koźmiński University - Lazarski University - Collegium Civitas in Warsaw - Maritime University of Szczecin - West Pomeranian University of Technology - University of Szczecin - WSB Merito University in Szczecin - Collegium Balticum in Szczecin - Jan Kochanowski University of Kilece - Kielce University of Technology - Nicolaus Copernicus University in Toruń - WSB Merito University in Toruń - University of Maria Curie-Skłodowska in Lublin - Lublin University of Technology - John Paul II Catholic University of Lublin - Polish Air Force University - University of Life Sciences in Lublin - University College of Enterprise and Administration in Lublin - WSEI University in Lublin - Łódź University of Technology - Rzeszów University of Technology - University of Rzeszów - University of Information Technology and Management in Rzeszów - University of Warmia and Mazury in Olsztyn - University of Finance and Information Technology in Kalisz - University of Zielona Góra - Białystok University of Technology - University of Białystok - Częstochowa University of Technology - University of Economics in Katowice | - [https://www.agh.edu.pl](http://www.agh.edu.pl/) - https://uek.krakow.pl - [https://www.pk.edu.pl](http://www.pk.edu.pl/) - [https://www.up.krakow.pl](http://www.up.krakow.pl/) - [https://www.awf.krakow.pl](http://www.awf.krakow.pl/) - [https://www.asp.krakow.pl](http://www.asp.krakow.pl/) - [https://www.amuz.krakow.pl](http://www.amuz.krakow.pl/) - [https://www.uj.edu.pl](http://www.uj.edu.pl/) - [https://www.wszib.edu.pl](http://www.wszib.edu.pl/) - https://upjp2.edu.pl - https://wse.krakow.pl - https://uksw.edu.pl - [https://www.sggw.edu.pl](http://www.sggw.edu.pl/) - [https://www.pw.edu.pl](http://www.pw.edu.pl/) - https://www.wojsko- polskie.pl/wat/ - https://asp.waw.pl - [https://www.sgh.waw.pl](http://www.sgh.waw.pl/) - https://chopin.edu.pl - [https://www.awf.edu.pl](http://www.awf.edu.pl/) - [https://www.uw.edu.pl](http://www.uw.edu.pl/) - [https://www.kozminski.edu.pl](http://www.kozminski.edu.pl/) - [https://www.lazarski.pl](http://www.lazarski.pl/) - https://civitas.edu.pl - [https://www.am.szczecin.pl](http://www.am.szczecin.pl/) - [https://www.zut.edu.p](http://www.zut.edu.pl/)l - https://usz.edu.pl - [https://www.merito.pl/szczecin/](http://www.merito.pl/szczecin/) - [https://www.cb.szczecin.pl](http://www.cb.szczecin.pl/) - [https://www.ujk.edu.pl](http://www.ujk.edu.pl/) - https://tu.kielce.pl - [https://www.umk.pl](http://www.umk.pl/) - [https://www.merito.pl/torun/](http://www.merito.pl/torun/) - [https://www.umcs.pl](http://www.umcs.pl/) - https://pollub.pl - [https://www.kul.pl](http://www.kul.pl/) - https://www.wojsko- polskie.pl/law/ - https://up.lublin.pl - https://wspa.pl - [https://www.wsei.lublin.pl](http://www.wsei.lublin.pl/) - https://p.lodz.pl - https://w.prz.edu.pl - [https://www.ur.edu.pl](http://www.ur.edu.pl/) - https://wsiz.edu.pl - https://uwm.edu.pl - https://wsfi.pl - https://uz.zgora.pl - https://pb.edu.pl - https://uwb.edu.pl - https://pcz.pl - https://www.ue.katowice.pl | March 29, 2023 |

# Supplementary Figures and Tables

## Supplementary Figures


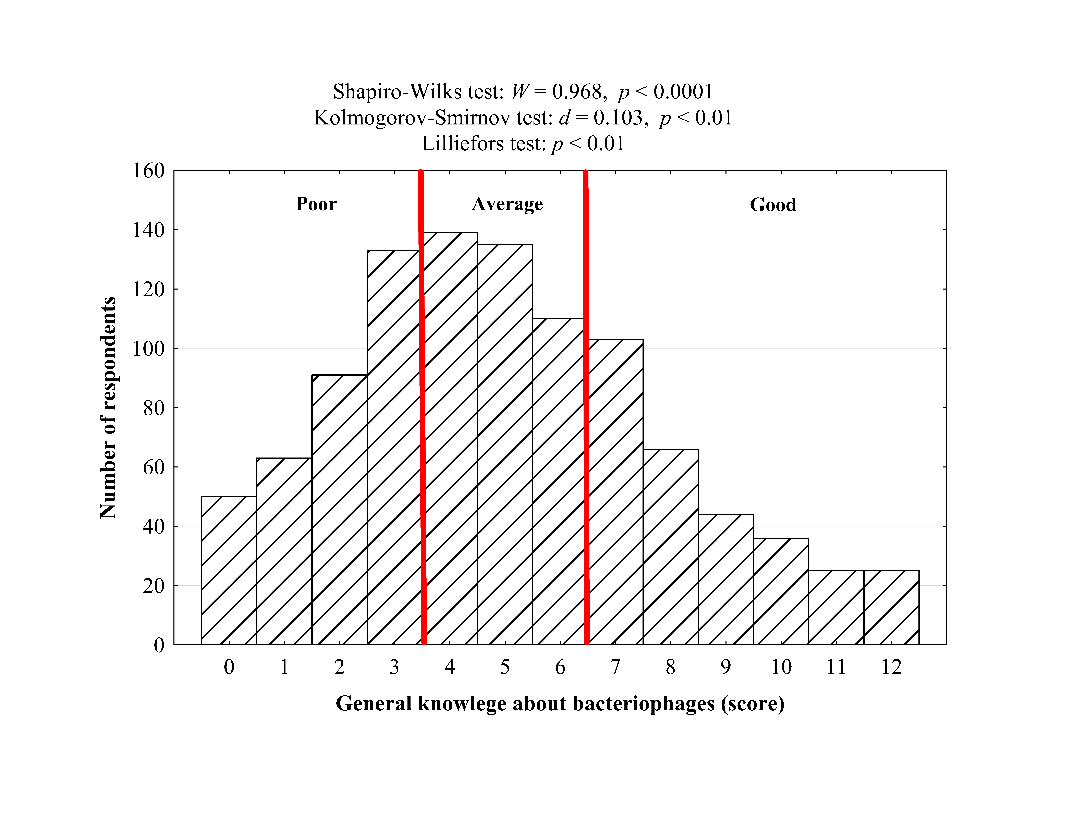


Supplementary Figure 1. Histogram of results of general knowledge about bacteriophages (assessed based on the GKB-12 scale) and their use in the treatment of antibiotic-resistant bacterial infections. Results of normality tests and the adopted division of respondents into three levels of knowledge.


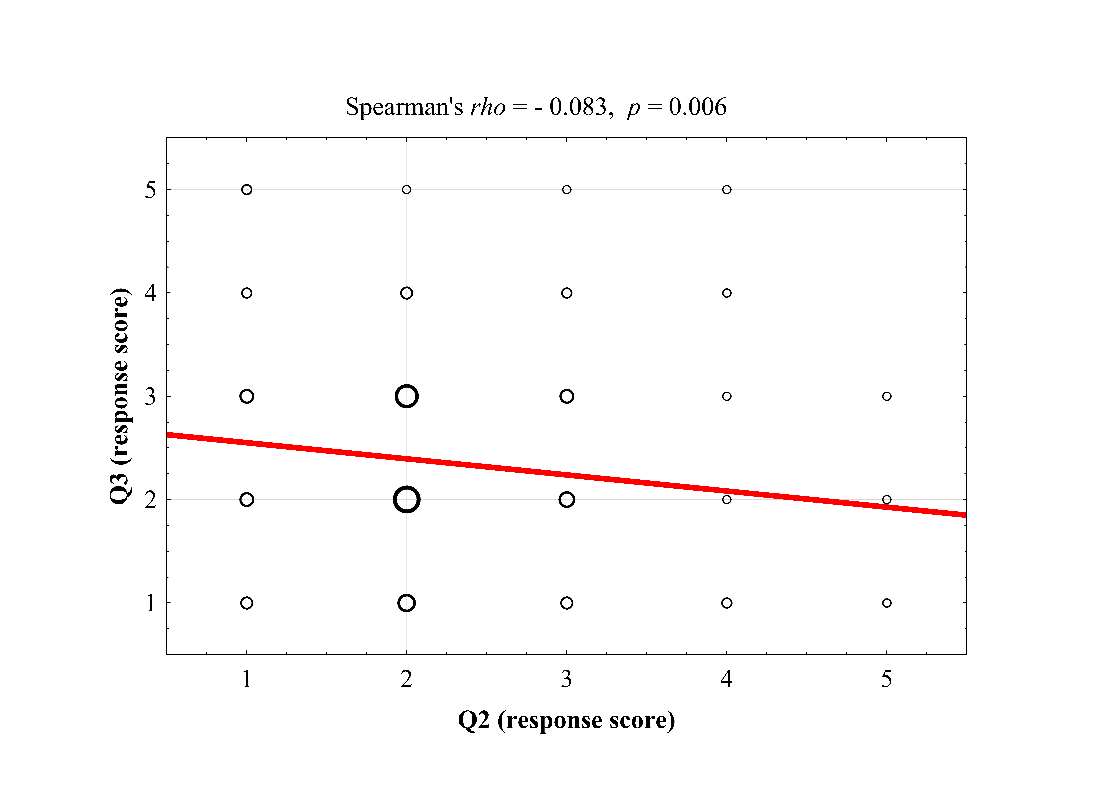


**Supplementary Figure 2.** Correlation diagram between answers to questions Q2 (In case of some diseases, when traditional forms of treatment provided by physicians are ineffective, experimental therapies conducted by them can lead to patient recovery) and Q3 (Alternative medicine offers better solutions to health problems than conventional medicine - i.e., based on reliable opinions and scientific research) and the value of Spearman's rank correlation coefficient. Scores were assigned to the responses as follows (both for Q2 and Q3): **1** - strongly disagree; **2** - I disagree; **3** - I neither agree, nor disagree, **4** - I agree; **5** - I strongly agree.


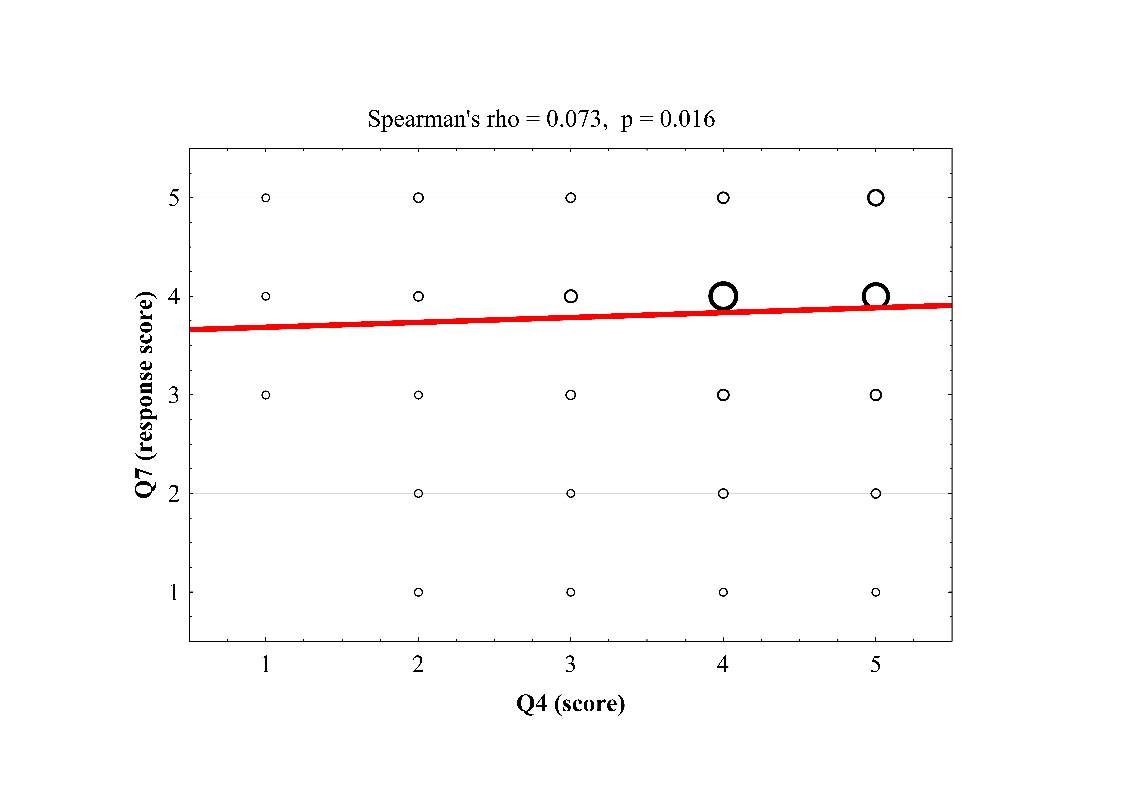


**Q4 (response score)**

**Supplementary Figure 3.** Correlation diagram between answers to questions Q4 (Before you take advantage of what alternative medicine offers, you must first try all the possibilities offered by conventional medicine) and Q7 (Do you think that in the case when there is a lack of effectiveness of standard treatment methods, it is worth trying experimental methods offered by academic medicine, even if their effectiveness is not confirmed?) and the value of Spearman's rank correlation coefficient. For Q4 scores were assigned to the responses as follows: 1 - strongly disagree; 2 - I disagree; 3 – I neither agree, nor disagree, 4 - I agree; 5 - I strongly agree. For Q7 scores were assigned to the responses as follows: 1 - definitely no; 2 - probably no; 3 - uncertain, 4 - probably yes; 5 - definitely yes.

**Supplementary Figure 4.** Scatter plots of GKB-12 scores depending on the answers to questions Q13 (Have you ever heard of bacteriophages before?) and Q14 (Have you ever heard of phage therapy?) and the regression line equation. For Q13 scores were assigned to the answers as follows: For Q13 scores were assigned to the responses as follows: 1 - No, I have never heard anything about it; 2 - Yes, I was familiar with this term, but I didn't know they were bacterial viruses; 3 - Yes, I knew before that bacteriophages are viruses that infect bacteria. For Q14 scores were assigned to the responses as follows: 1 - I have never heard of phage therapy and have no knowledge of it; 2 - I heard about phage therapy, but I did not know any details about how it works and who it is intended for; 3 - I heard about phage therapy and I know what it is.

**Q13 (response score) Q14 (response score)**


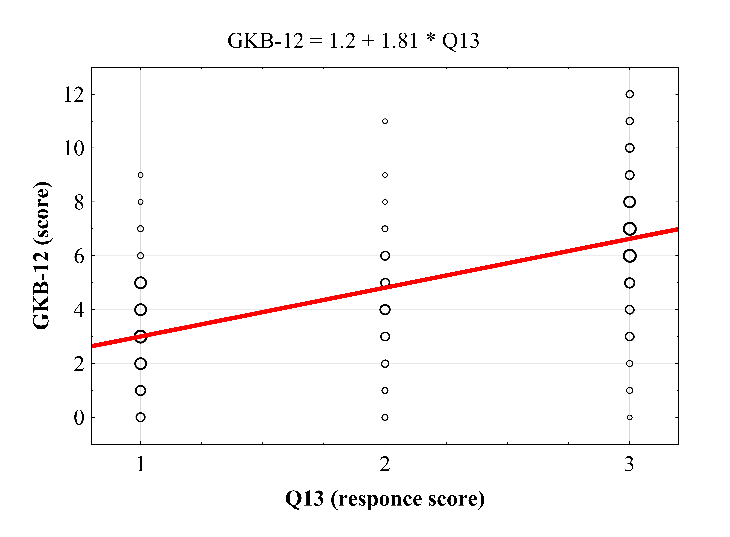

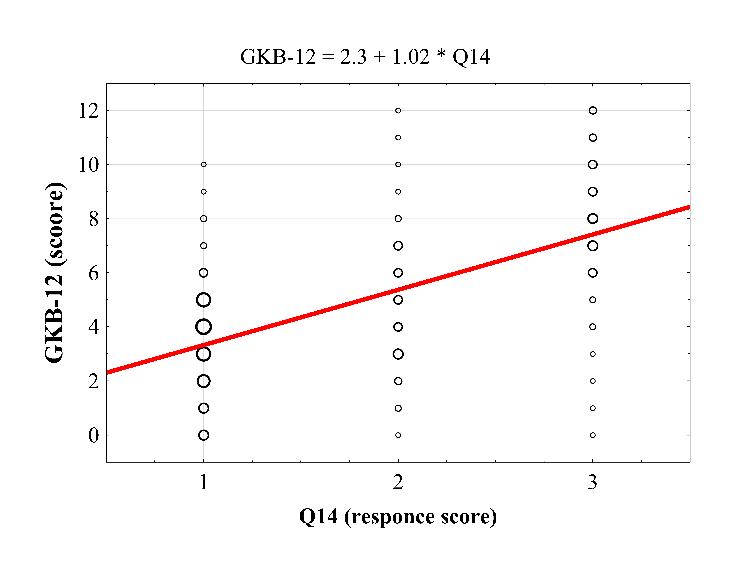


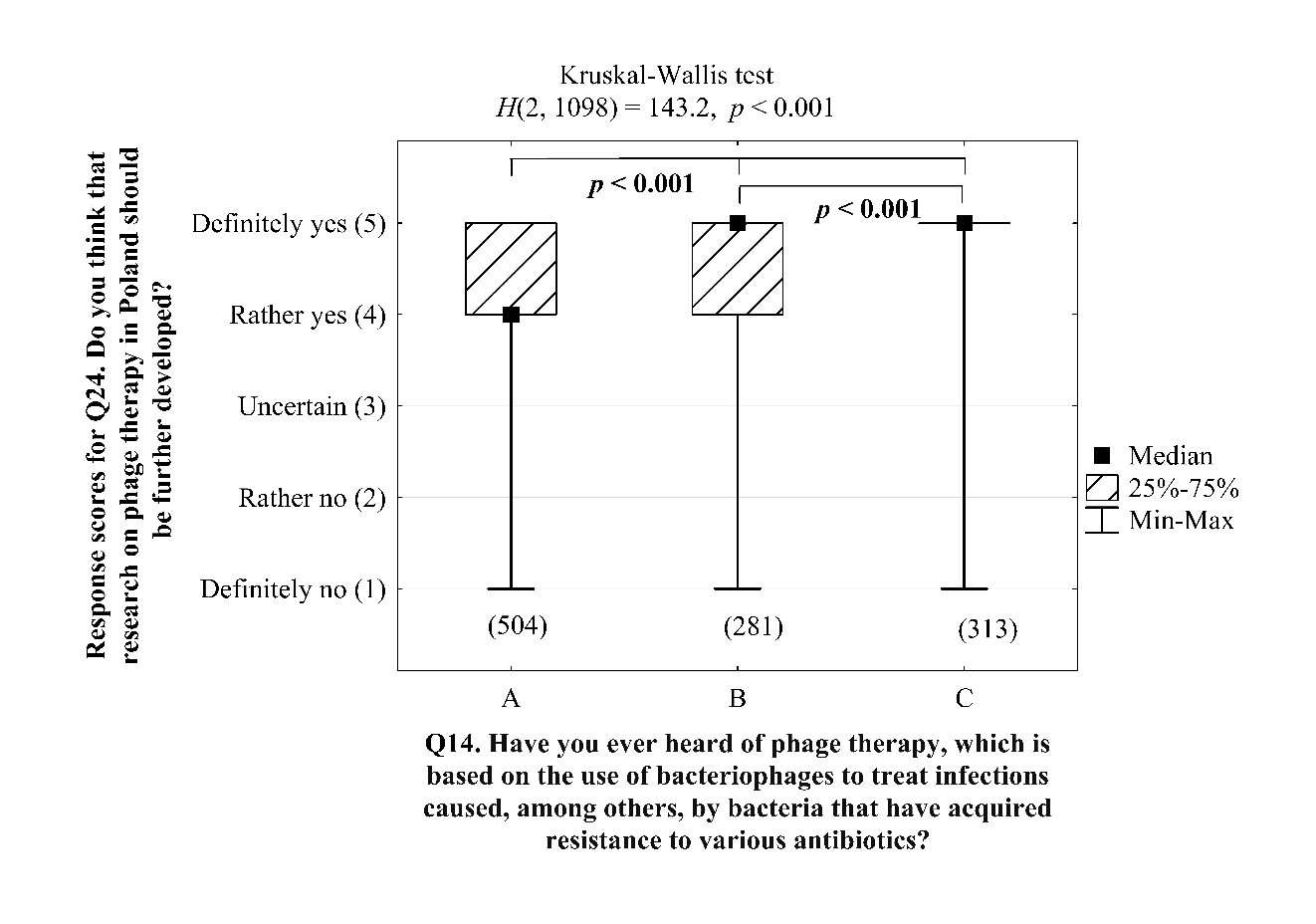


**Supplementary Figure 5.** Comparison of median scores for responses to Q24 in groups of respondents differing in their answers to Q14 and results of the Kruskal-Wallis significance test as well as *post-hoc* tests (Dunn's test). Answers to Q14 are as follows: A – I have never heard of phage therapy and have no knowledge of it; B – I heard about phage therapy, but I did not know any details about how it works; C – I heard about phage therapy and I know what it is. The sizes of the analyzed groups are given in brackets below columns and whiskers representing them.


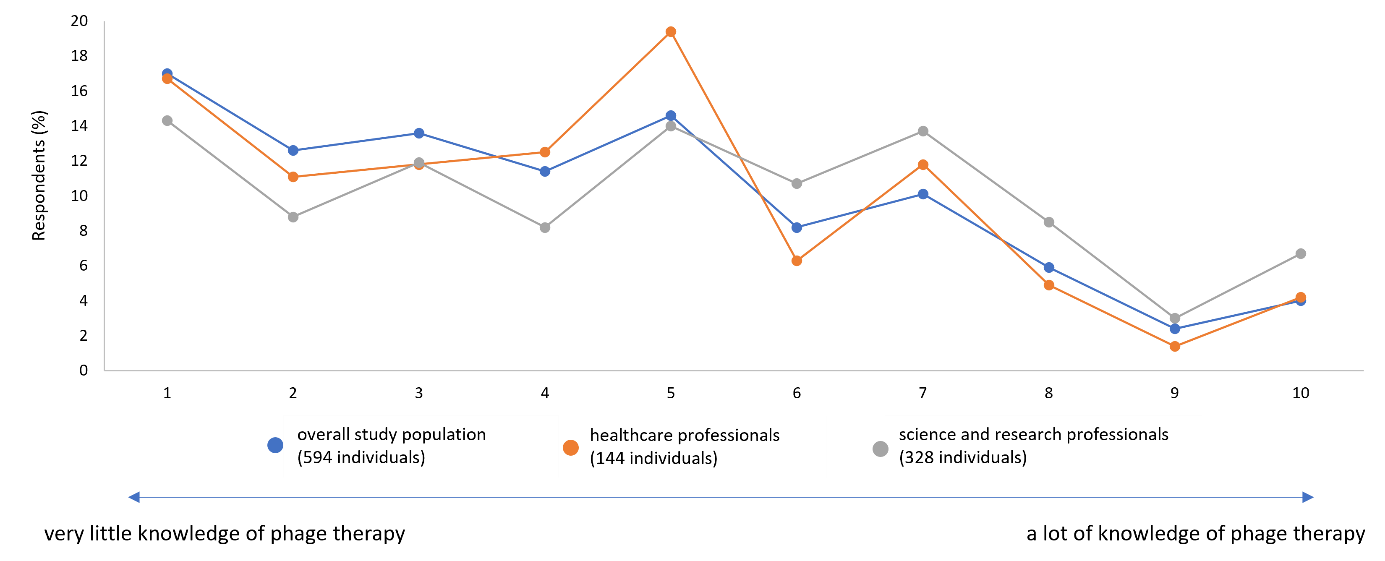


**Supplementary Figure 6.** Self-assessment of the level of knowledge about phage therapy (Q16) by the respondents who had heard of it.


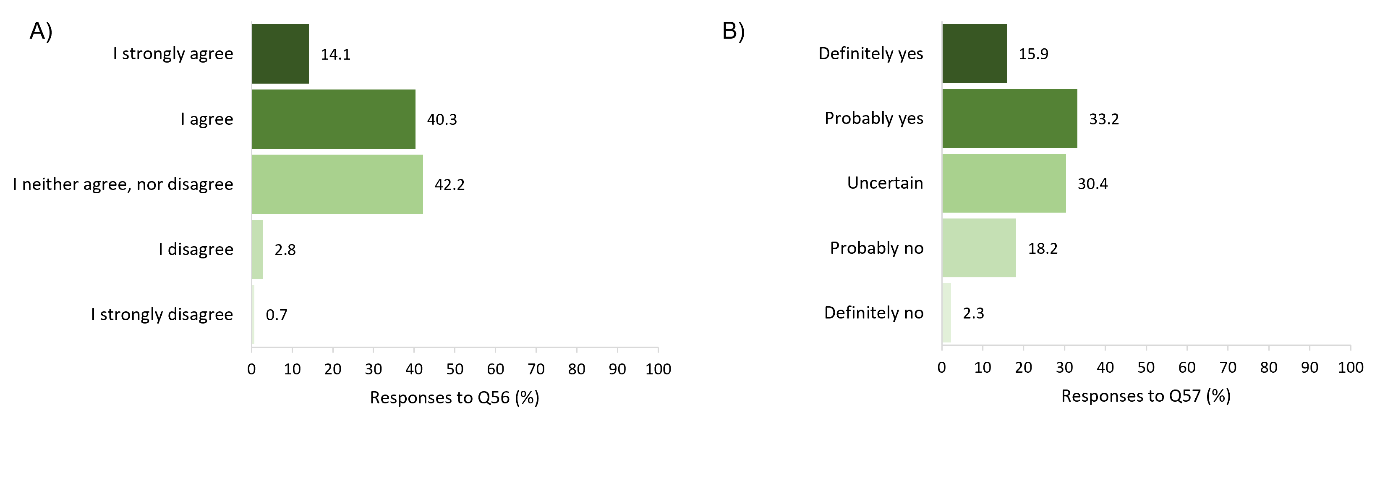


**Supplementary Figure 7.** Single-choice questions concerning phage treatment in humans (A) and phage treatment funding (B). Sample based on 434 responses. from the science and research sector. Questions are as follows: A) To what extent do you agree or disagree with the statement that phage therapy in humans is the most attractive and promising field of research on bacteriophages? (Q56); B) In your opinion, should public funds be increased for research on the therapeutic use of bacteriophages, even at the expense of other research fields? (Q57).


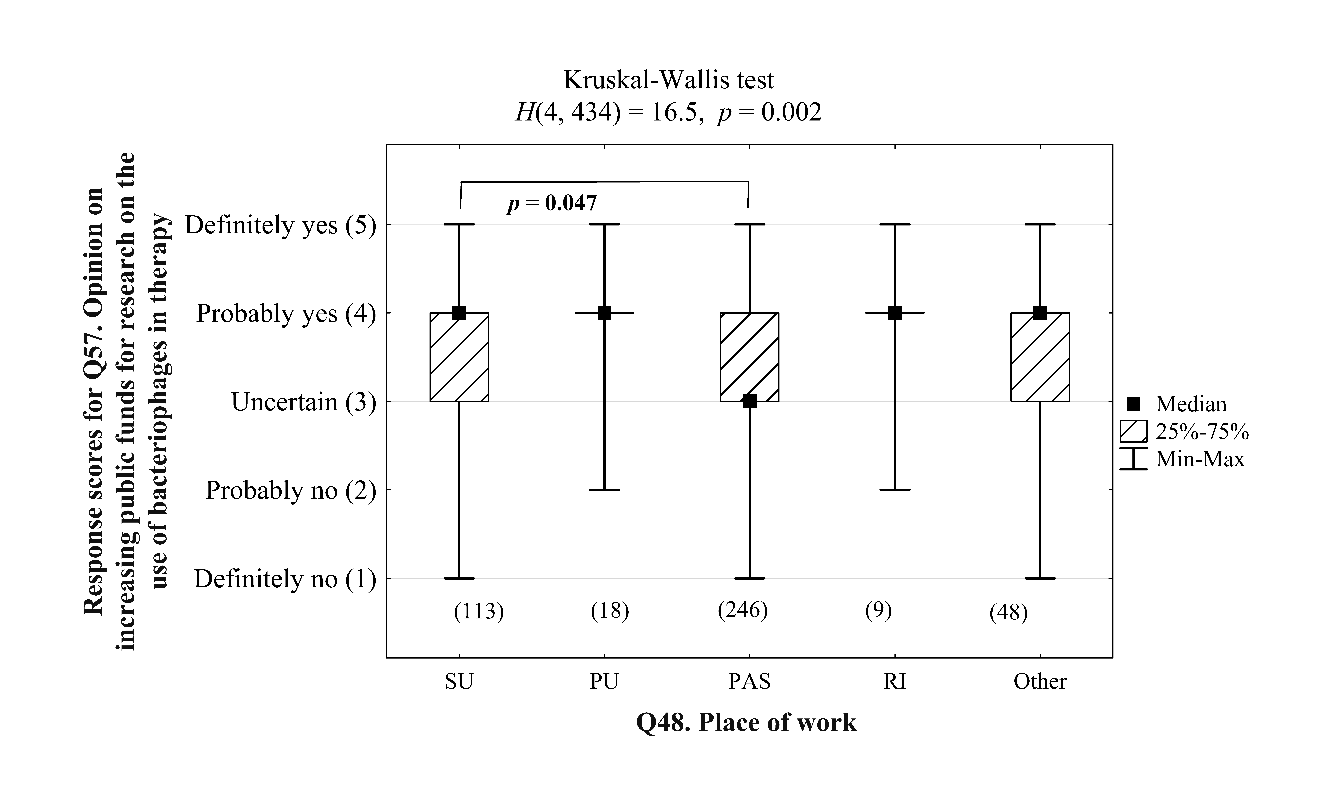


**Supplementary Figure 8.** Comparison of median scores for responses to Q57 regarding research financing in groups of science and research professionals depending on their place of work (SU - State university; PU - Private university; PAS - Institutes of the Polish Academy of Sciences; RI - Research institutes; O - Other research and scientific institutions) and the result of the Kruskal-Wallis significance test as well as *post-hoc* tests (Dunn's test). The sizes of the analyzed groups are given in brackets below columns and whiskers representing them.


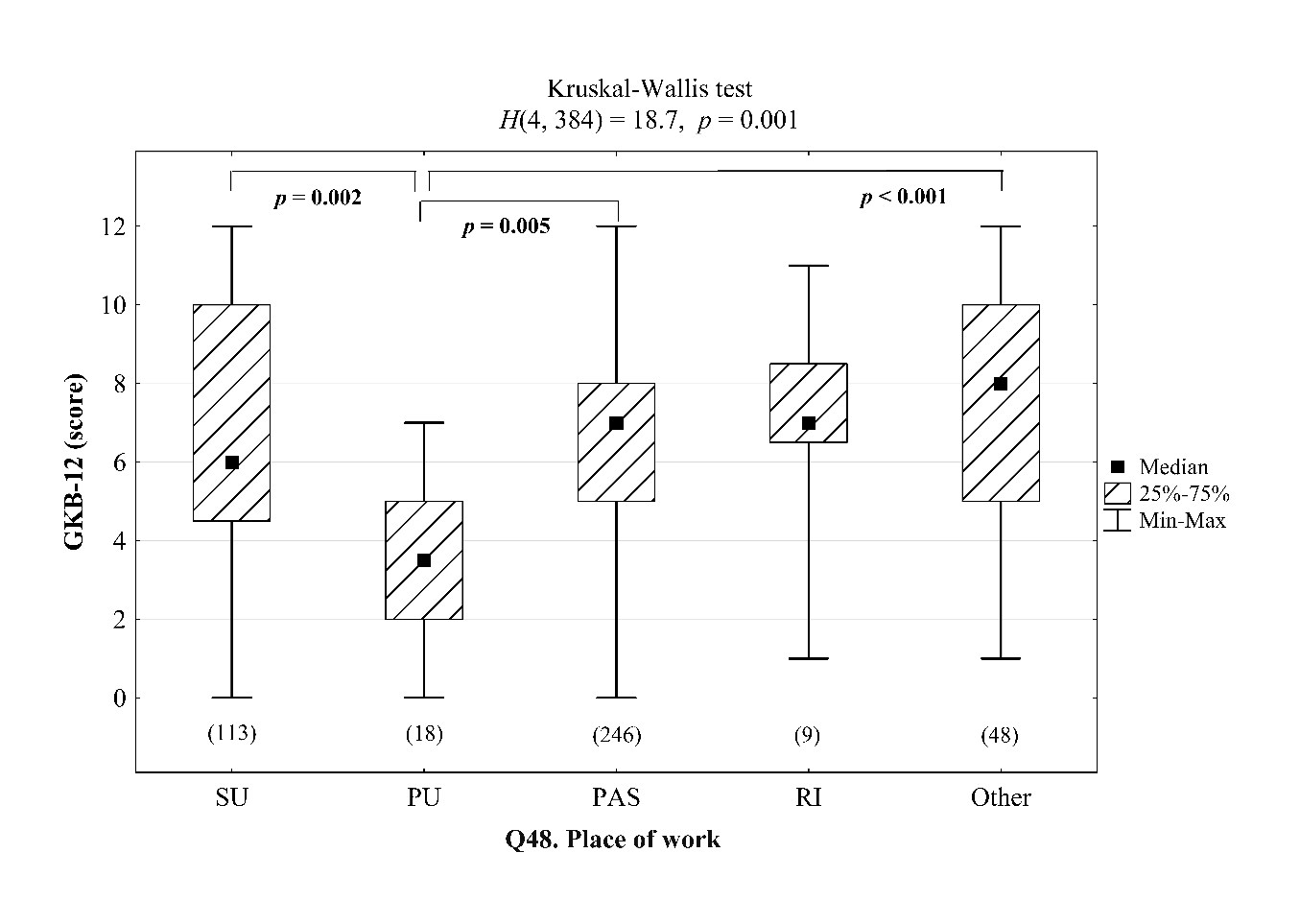


**Supplementary Figure 9.** Assessment of knowledge about bacteriophages on the GKB-12 scale in groups of science and research professionals depending on their place of work (SU - State university; PU - Private university; PAS - Institutes of the Polish Academy of Sciences; RI - Research institutes; O - Other research and scientific institutions) and the result of the Kruskal-Wallis significance test as well as *post-hoc* tests (Dunn's test). The sizes of the analyzed groups are given in brackets below columns and whiskers representing them.

## Supplementary Tables

**Supplementary Table 1.** Recruitment outcomes during the e-mail distribution period^a^

| **Organization/institution** | **Mailing date** | **Checking date** | **Number of participants**^b^ |
| --- | --- | --- | --- |
| Patients’ advocacy groups | October 25, 2022 | November 27, 2022 | F: 203  M: 93 |
| Institutes of the Polish Academy of Sciences | December 8, 2022 | January 26, 2023 | F: 354  M: 168 |
| Health care entities  (Round 1) | February 14, 2023 | February 28, 2023 | F: 371  M: 169 |
| Health care entities  (Round 2) | March 9, 2023 | March 24, 2023 | F: 436  M: 183 |
| Private and public universities (Round 1) | March 16, 2023 | March 24, 2023 | F: 436  M: 183 |
| Private and public universities (Round 2) | March 29, 2023 | April 17, 2023 | F: 496  M: 202 |

^a^ Applies only to *Arm A* (respondents reached by the authors)

^b^ First follow-up before initiation of email distribution (September 22, 2022): females (F) – 158, males (M) – 86; increase in the number of respondents over time corresponds with each mail distribution cycle

**Supplementary Table 2.** Design of analysis involving all questions and various examined groups of respondents.

| **Subject Matter** | **Question No.** | **Design of analysis** |
| --- | --- | --- |
| questions about the perception of experimental therapies, including clinical trials and COVID-19 pandemic  (first module) | 2-7 | - all answers summarized as frequencies and/or percentages |
| questions concerning the awareness and perception of antibiotic resistance  (first module) | 8-12 | - all answers summarized as frequencies and/or percentages - answers to Q11-12 summarized by the second and third module - answers to Q9 compared between selected groups and by awareness of phage therapy (Q13-Q14) |
| questions concerning the knowledge, perception of and experience with phage treatment  (first module) | 13-22; 24 | - all answers summarized as frequencies and/or percentages - answers to Q13-14 compared by sex, age, place of residence, level of education, employment status, profession, financial status, experience with health care and between two groups of participants (*Arm A* vs. *Arm B*) - answers to Q24 compared by awareness of phage therapy (Q14) |
| additional questions not directly related to key issues  (first module) | 23; 25-26 | - all answers summarized as frequencies and/or percentages |
| questions on population characteristics  (first module) | 1; 27-36 | - all answers summarized as frequencies and/or percentages |
| questions intended for health care sector (second module) | 37-47 | - all answers summarized as frequencies and/or percentage |
| questions intended for science and research sector (third module) | 48-57 | - all answers summarized as frequencies and/or percentages |
| optional quiz for everyone completing the questionnaire (fourth module) | 59-70 | - all answers summarized as frequencies and percentages |

Comment: question 1 was a question about gender; question 58 was an introduction to the quiz and included consent to participate in it.

**Supplementary Table 3.** The number (percentage) of respondents in subgroups differing in their answers to questions Q2 and Q3 and the result of the chi-square heterogeneity test.

| **Q3. Alternative medicine offers better solutions to health problems than conventional medicine** | **Q2. In case of some diseases, when traditional forms of treatment provided by physicians are ineffective, experimental therapies conducted by them can lead to patient recovery** | | | | | ***p*-value**^a^ |
| --- | --- | --- | --- | --- | --- | --- |
|  | I strongly agree | I agree | I neither agree nor disagree | I disagree | I strongly disagree |  |
| I strongly agree | 30 (69.8) | 8 (18.6) | 2 (4.7) | 3 (7.0) | 0 (0.0) | **< 0.001** |
| I agree | 21 (19.1) | 58 (52.7) | 25 (22.7) | 6 (5.5) | 0 (0.0) |  |
| I neither agree nor disagree | 63 (19.7) | 171 (53.4) | 77 (24.1) | 8 (2.5) | 1 (0.3) |  |
| I disagree | 61 (16.6) | 211 (57.5) | 82 (22.3) | 10 (2.7) | 3 (0.8) |  |
| I strongly disagree | 60 (23.3) | 115 (44.6) | 49 (19.0) | 24 (9.3) | 10 (3.9) |  |

**Supplementary Table 4**. The number (percentage) of respondents in subgroups differing in their answers to questions Q4 and Q7 and the result of the chi-square heterogeneity test.

| **Q4. Before you take advantage of what alternative medicine offers, you must first try all the**  **possibilities offered by conventional medicine** | **Q7. Do you think that in the case when there is a lack of effectiveness of standard treatment methods, it is worth trying experimental methods offered by academic medicine, even if their effectiveness is not confirmed?** | | | | | ***p*-value**^a^ |
| --- | --- | --- | --- | --- | --- | --- |
|  | Definitely no | Probably no | Uncertain | Probably yes | Definitely yes |  |
| I strongly disagree | 0  (0.0%) | 0  (0.0%) | 1  (0.7%) | 5  (0.9%) | 7  (2.9%) | **< 0.001** |
| I disagree | 2  (6.4%) | 10  (11.4%) | 12  (7.9%) | 28  (4.8%) | 27  (11.1%) |  |
| I have no opinion | 10 (32.3%) | 17  (19.3%) | 30  (19.7%) | 79 (13.5%) | 33  (13.6%) |  |
| I agree | 8 (25.8%) | 33 (37.5%) | 55  (36.2%) | 249 (42.6%) | 58  (23.9%) |  |
| I strongly agree | 11 (35.5%) | 28  (31.8%) | 54  (35.5%) | 223 (38.2%) | 118  (48.6%) |  |

**Supplementary Table 5**. Concerns related to the possible infection with antibiotic-resistant bacteria

| **Group of responders** | **Q12: How worried are you about** **getting infected with antibiotic-resistant bacteria?** | | | | | ***p*-value**^a^ |
| --- | --- | --- | --- | --- | --- | --- |
|  | Very afraid | Afraid | Neither afraid or unafraid | Unafraid | Very unafraid |  |
| Health care professionals  (n=195) | 12  (6.2%) | 33  (16.9%) | 57  (29.2%) | 58  (29.7%) | 35  (17.9%) | 0.051 |
| Rest of the population (n= 903) | 47  (5.2%) | 192 (21.3%) | 293  (32.4%) | 275  (30.5%) | 96  (10.6%) |  |
| Science and research professionals (n=434) | 22  (5.1%) | 81  (18.7%) | 140  (32.3%) | 142  (32.7%) | 49  (11.3%) | 0.570 |
| Rest of the population  (n=664) | 37  (5.6%) | 144  (21.7%) | 210  (31.6%) | 191  (28.8%) | 82  (12.3%) |  |

^a^ Pearson’s chi-square test

**Supplementary Table 6.** Number (percentage) of responses to question Q13 regarding awareness of the existence of bacteriophages in the lay people group (neither health care professionals nor science and research professionals) in Arm A *vs* Arm B.

| **Arm** | **Q13. Bacteriophages (also called phages) are bacteria-specific viruses. They infect them, multiply in them, and after multiplying they can destroy them. Have you ever heard of bacteriophages before?** | | | ***p*-value**^a^ |
| --- | --- | --- | --- | --- |
|  | No, I have never heard anything about it | Yes, I was familiar with this term, but I didn't know they were bacterial viruses | Yes, I knew before that bacteriophages are viruses that infect bacteria |  |
| Arm A  N = 185 | 65  (35.14%) | 47  (25.41%) | 73  (39.46%) | <0.001 |
| Arm B  N = 375 | 241  64.27% | 100  26.67% | 34  9.07% |  |

^a^ Pearson’s chi-square test

**Supplementary Table 7.** Number (percentage) of responses to question Q13 regarding awareness of the existence of bacteriophage therapy in the lay people group (neither health care professionals nor science and research professionals) in Arm A *vs* Arm B.

| **Arm** | **Q14. Have you ever heard of phage therapy, which is based on the use of bacteriophages to treat infections caused, among others, by bacteria that have acquired resistance to various antibiotics?** | | | ***p*-value**^a^ |
| --- | --- | --- | --- | --- |
|  | I heard about phage therapy and I know what it is | I heard about phage therapy, but I did not know any details about how it works and who it is intended for | I have never heard of phage therapy and have no knowledge of it |  |
| Arm A  N = 185 | 48  (25,95%) | 47  (25,41%) | 90  (48,65%) | <0.001 |
| Arm B  N = 375 | 15  (4,00%) | 91  (24,27%) | 269  (71,73%) |  |

^a^ Pearson’s chi-square test

**Supplementary Table 8.** Number (percentage) of responders who heard about phage therapy in school and/or college (response a for Q15). Data calculated for group of 594 responders who ever heard of phage therapy in Q14.

| **Answers provided** | **Age of responders (years)** | | | | | | | |
| --- | --- | --- | --- | --- | --- | --- | --- | --- |
|  | 15-18 | 19-24 | 25-34 | 35-44 | 45-54 | 55-64 | 65-74 | 75-91 |
| Yes | 10  (45.5%) | 42  (77.8%) | 108  (66.3%) | 76  (53.1%) | 33  (29.7%) | 15  (20.5%) | 4  (15.7%) | 0  (0.0%) |
| No | 12  (54.5%) | 12  (22.2%) | 55  (33.7%) | 67  (46.9%) | 78  (70.3%) | 58  (79.5%) | 20  (83.4%) | 4  (100.0%) |

**Supplementary Table 9.** Health care professionals who have encountered the use of bacteriophages in their work (Q41)^a^

| **Answers** | **Frequency** | | **Percentage** |
| --- | --- | --- | --- |
| No, because in my professional practice I do not meet people who suffer from difficult to treat bacterial infections | | 87 | 44.6% |
| No, although in my professional practice I meet people who suffer from difficult to treat bacterial infections | | 80 | 41.0% |
| Yes, but it was an isolated case | | 12 | 6.2% |
| Yes, more than once | | 16 | 8.2% |

^a^195 health care professionals

**Supplementary Table 10.** Most interesting topics as seen by the science and research professionals (Q53)^a,b^

| **Answers** | **Popularity**^c^ | | **Percentage** |
| --- | --- | --- | --- |
| Phage biology (occurrence of phages, their isolation, biological properties) | | 165 | 45.0% |
| Molecular biology of phages | | 152 | 41.4% |
| Therapeutic use of phages in the treatment of humans, animals and plants | | 308 | 83.9% |
| Manufacturing of phage preparations intended for therapy | | 189 | 51.5% |
| Bacteriophage activity unrelated to their antibacterial properties | | 138 | 37.6% |
| None of the above topics are of interest to me | | 7 | 1.9% |

^a^367 science and research professionals

^b^multi-choice question

^c^number of times the answer was selected

**Supplementary Table 11.** Number (percentage) of responses to the question regarding the perception of financing for phage research among science and research professionals (n=434).

| **Place of work (Q48)** | **Q57: In your opinion, should public funds be increased for research on the therapeutic use of bacteriophages, even at the expense of other research fields?** | | | | | ***p –* value^a^** |
| --- | --- | --- | --- | --- | --- | --- |
|  | **Definitely yes (5)** | **Probably yes (4)** | **I have no opinion (3)** | **Probably no (2)** | **Definitely no (1)** |  |
| A. State university | 27 (23.9%) | 36 (31.9%) | 30 (26.5%) | 18 (15.9%) | 2 (1.8%) | **0.021** |
| B. Private university | 3 (16.7%) | 12 (66.7%) | 0 (0.0%) | 3 (16.7%) | 0 (0.0%) |  |
| C. Institutes of the Polish Academy of Sciences | 28 (11.4%) | 73 (29.7%) | 87 (35.4%) | 51 (20.7%) | 7 (2.8%) |  |
| D. Research institute | 2 (22.2%) | 5 (55.6%) | 1 (11.1%) | 1 (11.1%) | 0 (0.0%) |  |
| E. Other | 9 (18.8%) | 18 (37.5%) | 14 (29.2%) | 6 (12.5%) | 1 (2.1%) |  |
| **Total** | **69 (15.9**%**)** | **144 (33.2**%**)** | **132 (30.4**%**)** | **79 (18.2**%**)** | **10 (2.3**%**)** |  |

^a^ Pearson chi-square
